# Phenotypic and Genetic Characterization of Avian Influenza H5N2 Viruses with Intra- and Inter-Duck Variations in Taiwan

**DOI:** 10.1371/journal.pone.0133910

**Published:** 2015-08-11

**Authors:** Yao-Tsun Li, Hui-Ying Ko, Chang-Chun David Lee, Ching-Yu Lai, Chuan-Liang Kao, Chinglai Yang, Won-Bo Wang, Chwan-Chuen King

**Affiliations:** 1 Institute of Epidemiology and Preventive Medicine, College of Public Health, National Taiwan University (NTU), Taipei, Taiwan, Republic of China (R.O.C); 2 Graduate Institute of Microbiology, College of Medicine, NTU, Taipei, Taiwan, R.O.C; 3 Department of Microbiology and Immunology, Emory University School of Medicine, Atlanta, Georgia, 30322, United States of America; St. Jude Children's Research Hospital, UNITED STATES

## Abstract

**Background:**

Human infections with avian influenza viruses (AIVs) have frequently raised global concerns of emerging, interspecies-transmissible viruses with pandemic potential. Waterfowl, the predominant reservoir of influenza viruses in nature, harbor precursors of different genetic lineages that have contributed to novel pandemic influenza viruses in the past.

**Methods:**

Two duck influenza H5N2 viruses, DV518 and DV413, isolated through virological surveillance at a live-poultry market in Taiwan, showed phylogenetic relatedness but exhibited different replication capabilities in mammalian Madin-Darby Canine Kidney (MDCK) cells. This study characterizes the replication properties of the two duck H5N2 viruses and the determinants involved.

**Results:**

The DV518 virus replicated more efficiently than DV413 in both MDCK and chicken DF1 cells. Interestingly, the infection of MDCK cells by DV518 formed heterogeneous plaques with great differences in size [large (L) and small (S)], and the two viral strains (p518-L and p518-S) obtained from plaque purification exhibited distinguishable replication kinetics in MDCK cells. Nonetheless, both plaque-purified DV518 strains still maintained their growth advantages over the plaque-purified p413 strain. Moreover, three amino acid substitutions in PA (P224S), PB2 (E72D), and M1 (A128T) were identified in intra-duck variations (p518-L vs p518-S), whereas other changes in HA (N170D), NA (I56T), and NP (Y289H) were present in inter-duck variations (DV518 vs DV413). Both p518-L and p518-S strains had the N170D substitution in HA, which might be related to their greater binding to MDCK cells. Additionally, polymerase activity assays on 293T cells demonstrated the role of vRNP in modulating the replication capability of the duck p518-L viruses in mammalian cells.

**Conclusion:**

These results demonstrate that intra-host phenotypic variation occurs even within an individual duck. In view of recent human infections by low pathogenic AIVs, this study suggests possible determinants involved in the stepwise selection of virus variants from the duck influenza virus population which may facilitate inter-species transmission.

## Introduction

Influenza has been a major concern for global health due to dynamic changes through continuous antigenic changes and occasional antigenic shifts resulting from the reassortment of viruses derived from different hosts [1]. Emerging novel influenza viruses, such as the 2006 H5N1 clade 2.2.1 virus in Egypt [2, 3], the 2009 swine-origin pandemic H1N1 virus [4], the 2011–2012 H3N2 variant in the United States [5], the 2013 H7N9 virus from human cases in China [6], and the first reported human H6N1 influenza pneumonia case in Taiwan [7], have highlighted the need for better understanding of the evolution and pathogenicity of avian influenza viruses (AIVs).

Routine virological surveillance in live-poultry markets (LPMs) has become imperative since the outbreaks of H5N1 influenza virus occurred in Hong Kong in 1997 [8]. Most importantly, the information on viral sequences provided clues to the origins of pathogens, and evaluation of inter-species transmission has great value in obtaining these molecular signatures for risk assessment and improved public health preparedness [9, 10]. Moreover, molecular and phenotypic characterizations of AIVs are critical for discovering new mechanisms and determinants involved in virus replication, virus-host interactions, host adaptation and immunopathology [11, 12].

Waterfowl, the major influenza virus reservoir, harbors genes that might contribute to AIVs with the potential to infect humans [12, 13]. Recent findings of H5N1 virus spread among ducks and other avian species suggest that ducks could also play an important role in influenza virus transmission [14, 15]. However, virological studies on dynamic changes in low pathogenic avian influenza (LPAI) viruses in ducks are few. To better monitor AIVs, we initiated a routine virological surveillance of influenza viruses in healthy ducks at a large, wholesale LPM in 2005 [16] and isolated LPAI H5N2 viruses for further characterization [17].

Here we report that the two Taiwan duck influenza H5N2 isolates, DV518 and DV413 [17], with high genetic sequence identities (nucleotides for eight viral genes ranging 99.6–100%) showed differences in their growth efficiencies in both mammalian and avian cells. In addition, heterogeneous viral sub-populations of LPAI viruses with different replication efficiency in the mammalian cells studied did exist within one individual duck influenza DV518 isolate. These findings could help understand the important elements in viral selection, pathogenesis and inter-species transmission from LPAI.

## Materials and Methods

### Sources of Taiwan duck influenza H5N2 viruses

Several Taiwan duck LAPI H5N2 viruses with different gene constellations that were isolated from fecal droppings in the environment of a live-poultry market of the previous study before 2012 [16] were examined [Surveillance protocol was approved by the Institutional Animal Care and Use Committee (IACUC) at National Taiwan University, Permit Number: 98–10]. Sequence analyses revealed the phylogenetic closeness between the two duck H5N2 viruses—DV518 and DV413 (i.e. parental viruses), with only three amino acid differences in all of the eight gene segments [17]. Therefore, these two virus strains were selected to examine their phenotypic variations in growth properties.

### Production of infectious viruses in cell culture

Madin-Darby Canine Kidney epithelial cells (MDCK) and chicken embryo fibroblast cells (DF1) [18] were grown at 37°C in Dulbecco’s Modified Eagle Medium (DMEM) (Invitrogen) or Minimum Essential Medium (MEM) (Invitrogen) supplemented with 10% fetal bovine serum (FBS) (Invitrogen), antibiotics and antimycotic reagents containing 100 μg/mL Streptomycin, 100 units/mL Penicillin and 0.25 μg/mL Amphotericin B (Invitrogen), respectively. For viral infection, serum-free media containing 1 or 0.05 μg/mL L-(tosylamido-2-phenyl) ethyl chloromethyl ketone-treated trypsin (TPCK-trypsin) (Thermo) were used during the infection of MDCK and DF1 cells, respectively.

### Quantitative RT-PCR to measure viral RNA

Viral RNA (vRNA) was extracted from the supernatant of infected cells by using the QIAamp Viral RNA Mini kit (Qiagen). The standard M-vRNA of influenza virus was transcribed *in vitro* from an M-gene-carrying plasmid, which was constructed as previously described [19]. The tested sample and ten-fold diluted standard of influenza virus M gene RNA (M-vRNA), in which purified RNA concentration was previously measured by spectrophotometer (General Electronic Company), were then reverse-transcribed by SuperScript III (Invitrogen), using Uni-12 primer [20] in parallel.

For quantitation, the tested samples were measured by quantitative RT-PCR for M vRNA. All cDNA samples were subjected to real-time PCR using the KAPA SYBR FAST qPCR kit (KAPA) with AMF (5’-GAGTCTTCTAACCGAGGTCGAAACGTA -3’) and FluA-R (5’-CAAAGCGTCTACGCTGCAGTCC-3’) primers [19]. Standard curves were constructed by cycle threshold [*C*_*T*_] values of the diluted M-vRNA standard with known copy numbers.

### Plaque assay and plaque-purification of the viruses

The viral stock was first titrated by plaque assay using MDCK cells. Briefly, MDCK cells were seeded on six-well plates and grown until confluent. Ten-fold serial dilutions of the tested samples were performed in duplicate. After one-hour adsorption at 37°C, the inoculum was then removed and a 1:1 mixture of 1% agarose gel and 2X serum-free DMEM with TPCK-trypsin at a final concentration of 2 μg/mL was immediately added as an overlay medium. After 72 hours’ incubation at 37°C, the infected cells were fixed with paraformaldehyde and stained with crystal violet. For plaque-purification, approximately 5–10 plaques were picked after 72 hours incubation without fixing the cells and then individually inoculated into SPF eggs. The p518-L and p518-S virus strains were isolated from large and small plaques, respectively through three rounds of plaque purification from the DV518 virus stock, whereas p413 virus was isolated through only one round of plaque purification from the DV413 virus stock for further virological characterization. Experiments for different virus strains were conducted independently to prevent possible cross-contamination. Plaque morphology was examined after each round of plaque purification; and the sizes and numbers of plaques in one well were analyzed by software Image J 1.45A (Wayne Rasband, National Institutes of Health, U.S.A.)

### Viral titration by 50% tissue culture infective dose (TCID_50_)

The TCID_50_ method was modified as previously described [21]. The 96-well plates were seeded with 1.5 x 10^4^ MDCK cells per well, and the tested samples were half-log serially diluted by medium containing TPCK-trypsin with at least four repeats. The virus was adsorbed at 37°C for two hours, ending with removal of the inoculum by washing twice. After 72 hours’ incubation at 37°C, the culture supernatant was harvested to determine the occurrence of infection by standard hemagglutination assay [22] [i.e. hemagglutination-positive]. Titers were calculated by the Reed-Muench method [23].

### Viral sequence analyses

All the studied viruses, from parent stocks to the plaque-purified strains generated during each round of the plaque-purification process, were used for full-genome sequencing. RNA extraction and reverse transcription were performed to obtain cDNA as mentioned above, using the influenza A virus Uni-12 primer [20]. Finally, cDNA was amplified by conventional PCR with a Platinum *Tag* DNA polymerase kit (Invitrogen). All the PCR products were sequenced by the Sanger method and then the sequence data was analyzed, using software of DNAMAN 7 (Lynnon Corp.) or SeqMan in Lasergene package (DNASTAR). Amino acid residues are shown with the H5 numbering system.

### Cell binding assay

MDCK and DF1 cells were grown to monolayer in 12-well plates. These two types of cells were then infected with the same copies per cell of the tested viruses, and then incubated at 4°C for one hour. The infected cells were washed with phosphate buffered saline (PBS) to remove unbound virions. Subsequently, Trizol (Invitrogen) was added to extract RNA from infected cells and the RNA was further extracted using the Direct-zol RNA MiniPrep kit (Zymo). The vRNA was quantified by quantitative RT-PCR.

### Minigenome assay

Before transfection, human kidney 293T cells were seeded onto 24-well plates and grown until semi-confluent. The cells were then co-transfected with the plasmid pPOL I-Luc-RT, which contains the luciferase open reading frame in negative polarity flanked by the noncoding regions of the NS gene of influenza A/WSN/33 virus [24], and the Renilla-luciferase-expression plasmid pRL-CMV (which serves as a transfection efficiency control) as described [24]. These cells were incubated at 37°C for 24 hours, and then were infected with the tested Taiwan duck H5N2 virus strains at an MOI of 2. The infected cells were harvested at 9 hours post-infection (h p.i.) as described [25], and the luciferase activity was assayed using a dual luciferase assay reagent kit (Promega).

### Western blotting

The 293T cells were seeded in six-well plates and grown until semi-confluent. To measure the infection efficiency, cells were infected with the tested p518-L and p518-S strains in serum-free medium without trypsin) at an MOI of 2. At 0.5, 1.5, or 6 h p.i., cell lysates were collected with modified radio-immuno-precipitation assay (RIPA) buffer supplemented with dithiothreitol (DTT) (Sigma) and protease inhibitors (Millipore). Protein content was determined by the Bradford method, using Bio-Rad Protein Assay kit (Bio-Rad). Proteins in these cell lysates were separated by 10% sodium dodecyl sulfate polyacrylamide gel electrophoresis (SDS-PAGE) and transferred to a polyvinylidene fluoride (PVDF) membrane. The membranes were incubated with rabbit anti-influenza NP antibodies (GeneTex) and mouse anti-Actin antibody (Millipore), and then stained with horseradish peroxidase (HRP)-conjugated secondary antibodies specific for mouse (Jackson) and rabbit (Cell Signaling). The membranes were developed with the enhanced chemiluminescence system (Perkin Elmer), and the images were obtained by the BioSpectrum Imaging System (UVP).

### Statistical analysis

Unpaired Student’s *t*-tests were conducted for statistical analyses by using an R program [26].

### Nucleotide sequence accession numbers

The nucleotide sequences of the two Taiwan duck H5N2 DV518 and DV413 virus strains obtained in the present study are available from GenBank under accession numbers KP792297-KP792312.

### Ethics statement

This research used only cell lines to investigate viral properties. The sources of the three cell lines used in this study, including (1) chicken embryo fibroblast DF1 cells (ATCC CRL-12203), (2) Madin-Darby Canine Kidney epithelial (MDCK) cells (ATCC CCL-34), and (3) human kidney 293T cells (ATCC CRL-3216) were kindly provided by Dr. Ching-Ho Wang, Dr. Chuan-Liang Kao, and Dr. Shiou-Hwei Yeh at the National Taiwan University, respectively. They were all bought from the American Type Culture Collection (ATCC), Rockville, Maryland, USA (http://www.atcc.org). The protocol of surveillance was approved by the Institutional Animal Care and Use Committee (IACUC) at the National Taiwan University [Permit Number: 98–10].

There was no gain of function performed in this study. We also confirm that the funders [National Institutes of Health (NIH) in the USA and the National Science Council [NSC, renamed as the Ministry of Science and Technology (MOST) since March of 2014 in Taiwan, Republic of China] had no role in study design, data collection and analysis, decision to publish or preparation of the manuscript.

## Results

### Growth kinetics of the two Taiwan duck H5N2 virus strains in MDCK and DF1 cells

**Fig 1:**
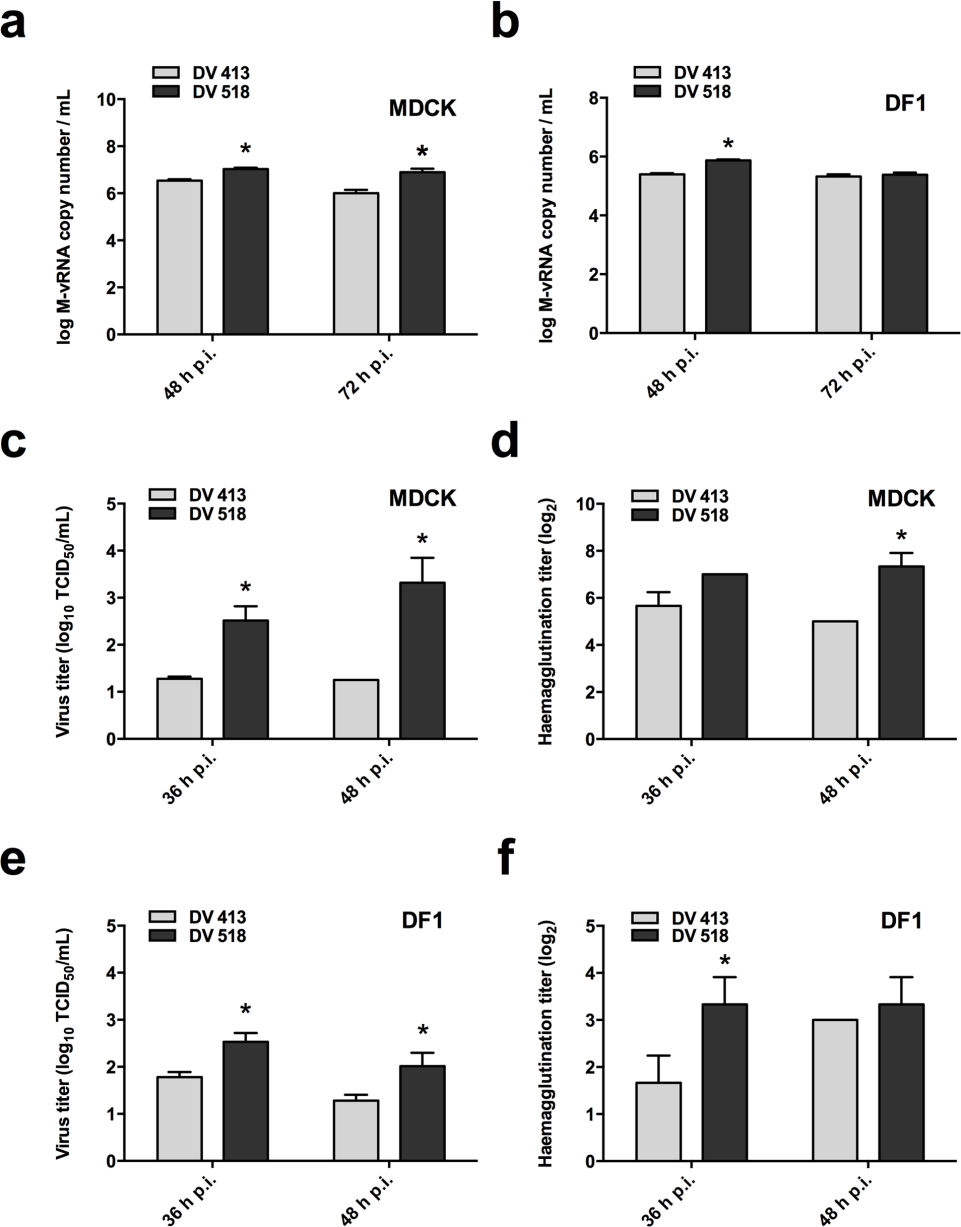
Growth properties of the two Taiwan H5N2 duck viruses in MDCK and DF1 cells. Levels of M-vRNA in the supernatant of (a) MDCK and (b) DF1 cells infected with DV518 and DV413 at MOI of 0.01 and 0.1, respectively, were measured by quantitative RT-PCR. In addition, the viral replication capabilities of DV518 and DV413 in MDCK and DF1 cells at MOI of 0.1 were evaluated by the TCID_50_ method (c and e) and the hemagglutination assay (d and f). Supernatant samples from the infected cells were harvested at the indicated time points. The results are from two independent experiments, presented as means ± standard deviations of triplicate samples. **Abbreviation: MOI**: multiplicity of infection; **TCID**_**50**_: 50% tissue culture infectious dose.*p<0.05

To investigate the replication levels of DV518 and DV413 *in vitro*, we first used quantitative RT-PCR to quantify the vRNAs in both mammalian and avian cell culture supernatants. Fig 1A shows that the MDCK cells infected with DV518 at an MOI of 0.01 generated significantly higher levels of vRNA after 48 h p.i. and 72 h p.i. when compared with those cells infected with DV413 [Mean: 10^7.03^ vs 10^6.54^ copies/mL (p<0.05) at 48 h p.i. and 10^6.90^ vs 10^6.01^ copies/mL (p<0.05) at 72 h p.i.]. In addition, chicken DF1 cells infected with these two virus strains at an MOI of 0.1, also showed quite similar results, with DV518 producing a significantly higher level of vRNA at 48 h p.i. than DV413 (Mean: 10^5.87^ vs 10^5.39^ copies/mL, p<0.05) (Fig 1B).

Besides vRNA levels, infectious virus titers were also quantified by the TCID_50_ method. MDCK cells infected with DV518 at a MOI of 0.1 resulted in significantly higher viral yield at 36 and 48 h p.i. when compared with the same cells infected with DV413. [Mean: DV518 vs DV413: 10^2.51^ vs 10^1.28^ TCID_50_/mL (p<0.05) at 36 h p.i. and 10^3.32^ vs 10^1.25^ TCID_50_/mL (p<0.05) at 48 h p.i.] (Fig 1C). Hemagglutination titers also revealed results similar to those of the TCID_50_ method in MDCK cells (Fig 1D). We also used the TCID_50_ and hemagglutination methods to quantify virus titers produced in DF1 cells. As shown in Fig 1E and 1F, DV518 produced significantly higher virus titers than DV413 in DF1 cells. Together, these data indicate that DV518 and DV413 differed in their growth in MDCK and DF1 cells, and the DV518 strain produced a higher virus yield.

### DV518 virus showed heterogeneous plaque sizes upon infection of MDCK cells

**Fig 2:**
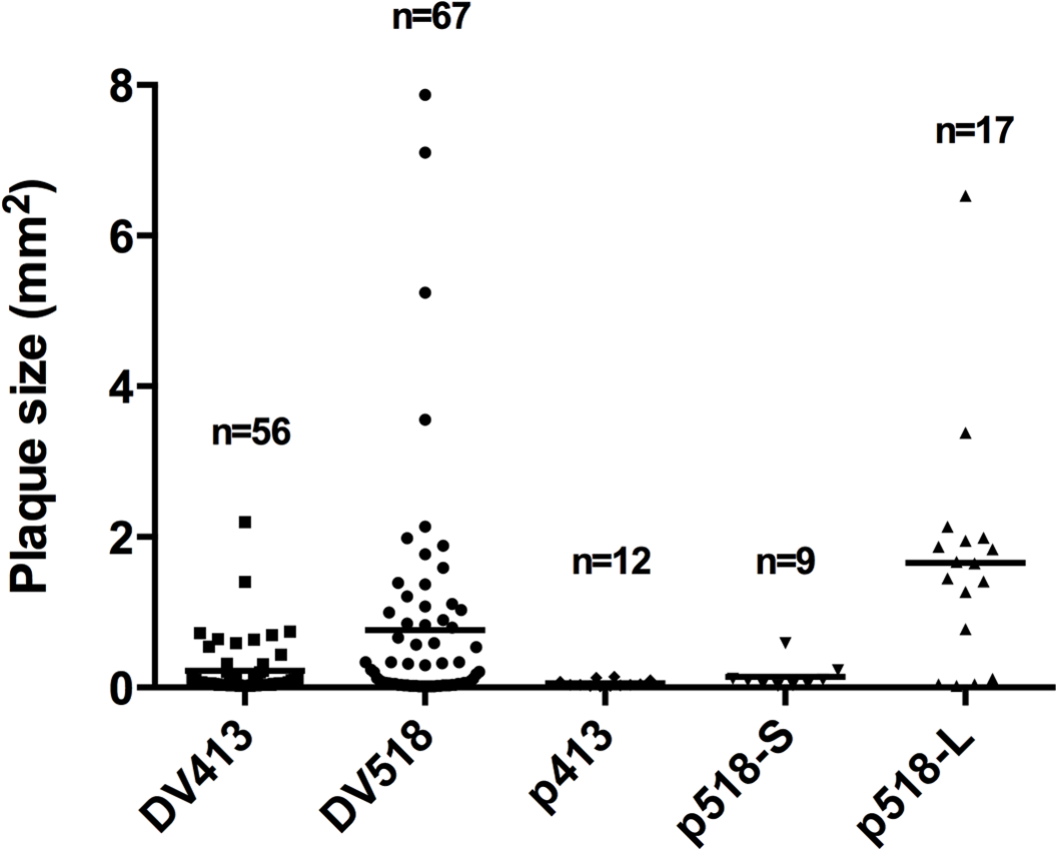
The distributions of plaque sizes in MDCK cells infected with DV518 and DV413 virus strains and the three plaque-purified virus strains. Plaque sizes (mm^2^) and numbers of plaques were analyzed by software Image J 1.45A in a representative well infected by DV518 and DV413 virus strains and their plaque-purified strains (p518-L, p518-S, and p413). Mean is shown by a parallel bar, and the total number of plaques is shown at the top, marked as n = 56, 67, 12, 9, and 17, respectively.

In plaque assays of DV518 and DV413 using MDCK cells, different-sized plaques were observed, implying these duck isolates could be a mixture of heterogeneous viral sub-populations. To test this, we analyzed the distribution of viral plaque sizes of DV518 and DV413 in MDCK cells. The results revealed that the duck DV518 strain generated plaques that were larger and more heterogeneous in size than those generated by the DV413 strain (S1 Fig). Fig 2 shows the distributions of plaque sizes of DV518 and DV413 strains in representative wells. The DV518 strain produced about 25% of the plaques that were larger than 1 mm^2^, whereas the DV413 strain had very few plaques larger than 1 mm (< 3% in Fig 2). In addition, about 25% of both DV518 and DV413 plaques ranged from 0.02 to 0.04 mm^2^ in area. These results indicate that the duck DV518 strain was a mixture of heterogeneous viral sub-populations. To further explore this hypothesis, we plaque-purified viruses from large (>1 mm^2^) and small plaques in DV518-infected MDCK cells (which were named p518-L and p518-S, respectively) as well as the plaque in DV413-infected MDCK cells (named p413) and then compared their growth properties *in vitro*.

Further analysis of plaque sizes of these purified viral strains (Fig 2 and S1 Fig) showed that the plaques generated by p518-L were larger than those generated by p518-S in MDCK cells (mean area 1.5 mm^2^ versus 0.3 mm^2^). In addition, the results from the focus-forming assay also verified the above findings (S2 Fig). However, the plaques generated by the p518-S strain were still larger than those plaques (which were pinhead-sized) generated by the p413 strain. Plaques generated by these purified virus strains were also more homogenous in size, although there was a wider standard deviation (SD) of p518-L, apparently influenced by the outliers (1.35±0.94 mm^2^ for p518-L without one outlier, n = 16).

### The plaque-purified duck H5N2 p518-L and p518-S virus strains exhibited different growth profiles in MDCK cells

**Fig 3:**
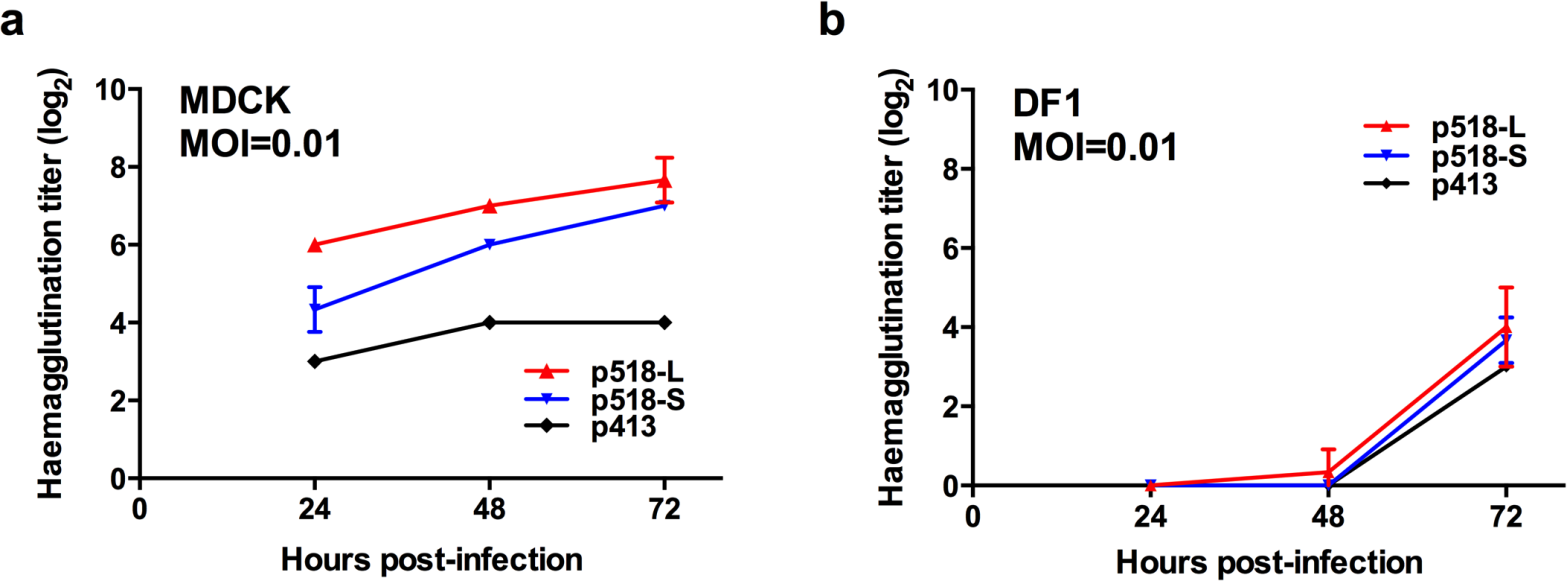
The growth kinetics of the three plaque-purified virus strains (p518-L, p518-S and p413) in MDCK and DF1 cells. MDCK and DF1 cells were infected with p518-L, p518-S, and p413 virus strains at an MOI of 0.01. The supernatants were harvested at the indicated time points. The hemagglutination assays were used to examine the viral growths. The results are shown as means ± standard deviations of triplicate samples. **MOI**: multiplicity of infection

The growth kinetics of these three plaque-purified virus strains (p518-L, p518-S and p413) were simultaneously examined in MDCK and DF1 cells using the hemagglutination method. As shown in Fig 3A, while p518-L produced the highest viral titers, p413-S had the lowest titers in MDCK cells. However, in DF1 cells, these purified viral strains produced comparable, but low, virus titers (Fig 3B). We also used the TCID_50_ method to measure the virus titers produced in MDCK cells. As shown in S3 Fig, p513-L and p513-S showed much higher titers than p413 the titers of which were under the detection limit by using the TCID_50_ method. It should be noted that the TCID_50_ method measures the viruses that can cause a cytopathic effect in MDCK cells. Thus the TCID_50_ method gave much lower titers for those virus strains with weak multiplication efficiency (i.e. p413) than those results obtained from the quantitative RT-PCR and the hemagglutination methods, which measure the amount of virus directly.

**Fig 4:**
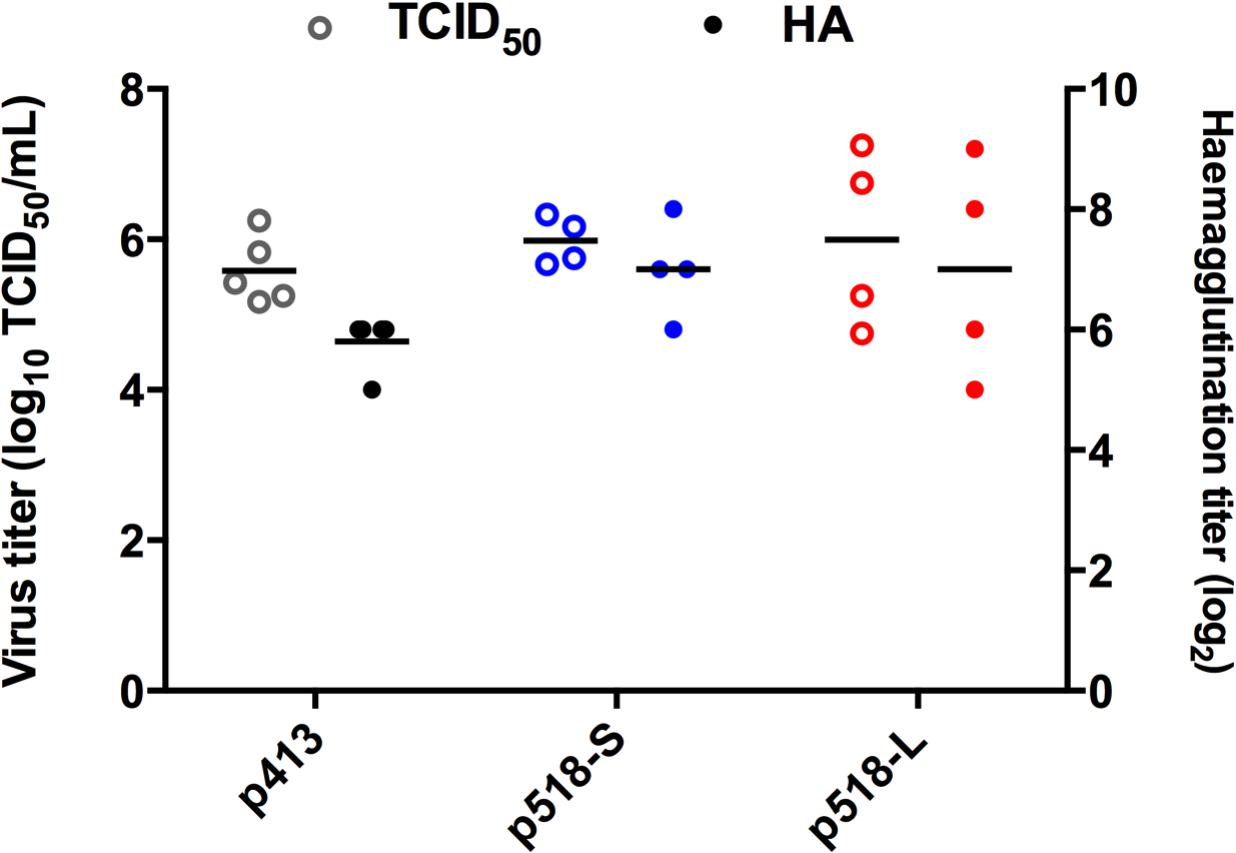
Viral yield in chicken embryonated eggs inoculated with p518-L, p518-S, and p413 virus strains. Five SPF eggs were inoculated with 4x10^5^ TCID_50_ titer of each of the p518-L, p518-S, and p413 virus strains. Eggs were euthanized at 48 h p.i. and their allantoic fluid was collected for measurement of viral load by either TCID_50_ method (empty circles "ο”) or hemagglutination assay (solid circles “•”). Mean values are shown by parallel bars. Both p518-S and p518-L inoculated groups had one uninfected egg (hemagglutination titer <1), which was excluded from the analyses. **SPF:** Specific pathogen free. **TCID**_**50**_ **:** 50% tissue culture infectious dose.

To verify the above findings from DF1 cells, we used an *in ovo* system with 14-day-old embryonated SPF eggs to mimic selective pressure for AI viruses in chickens [27, 28]. These three plaque-purified virus strains were inoculated into SPF eggs at 5,000 TCID_50_ units, and the virus yield in allantoic fluid at 48 h p.i. was examined. As shown in Fig 4, p518-L and p518-S grew to titers slightly higher than p413 (the peaking titers of p518-L, p518-S and p413-inoculated eggs were 10^7.25^, 10^6.3^, and 10^6.25^ TCID_50_/mL, respectively), although the differences did not reach statistical significance. This result is consistent with the data shown in Fig 3B. The data also indicate that p413 was able to replicate at low levels in DF1 cells, while it had limited ability to replicate in MDCK cells.

### Full-genome amino acid sequence analyses of the three plaque-purified H5N2 virus strains (p518-L, p518-S, and p413) and their parental virus strains (DV518 and DV413)

To gain more information on the mechanism involved in phenotypic variations of the plaque-purified virus strains, we sequenced p518-L, p518-S and p413 strains and compared the results with those obtained from the original viral isolates (marked as “original” DV518 and DV413 in Table 1). The original DV518 and DV413 viral stocks were obtained by amplification of the original fecal samples grown in the SPF chicken eggs. Therefore the sequence of DV518 and DV413 most likely represent the sequence of the major viral population in the original fecal samples. Table 1 summarizes the deduced amino acid variations. The purified p518-L and p518-S had the same signature at three amino acid residues (HA[170Asp], NA[56Thr], NP[289His]) as the original DV518 strain. In contrast, both original DV413 and purified p413 strains had different amino acids of HA[170Asn], NA[56Ile], and NP[289Tyr] at these positions (Table 1). In addition, p518-L possessed another two distinct variations in PB2 and M1 proteins (PB2[72Asp], M[182Thr]), compared with the residues at the respective positions shared by both p518-S and p413 strains (PB2[72Glu], M[182Ala]). On the other hand, p518-S had one residue (Pro) in PA at position 224 that was different from p413 and p518-L (PA[Ser224]). It is noteworthy that this PA[224Pro] variation is the only residue where p518-S differed from the original DV518 isolate (PA[Ser224]). Since p518-L and p518-S were obtained by triple plaque purification steps, we also sequenced their original plaques picked up during the first round of purification. The results showed that all molecular signatures described above were also present in the respective original plaques (without double base peaks in the sequence chromatogram), implying that the substitutions we detected did not emerge through selection during the purification procedure (see details in S4 Fig).

**Table 1:** Amino acid changes detected in eight gene segments of the DV518 and DV413 strains.

Virus	Amino acids at the following positions:					
	PB2	PA	HA	NP	NA	M1
	72	224	170	289	56	182
**518-Origin**	E	S	**D**	**H**	**T**	A
**p518-S**	E	**P**	D	H	T	A
**P518-L**	**D**	S	D	H	T	**T**
**413-Origin**	E	S	**N**	**Y**	**I**	A
**p413**	E	S	N	Y	I	A

**Sources of information:** Data were acquired from viral sequences of the two Taiwan duck H5N2 DV518 and DV413 virus strains in 2006 [17]

Both **518-Origin** and **413-Origin** of Taiwan duck influenza H5N2 viruses represent the viral sequences that were performed directly on the 1^st^ passage of egg culture from the duck fecal droppings that we collected from the floor of a live-poultry market.

**p518-L, p518-S:** The two virus strains were purified from either large or small “plaques” (with “p” added in the front) after the infection of DV518 in MDCK cells.

**Boldface amino acids** represent the residue differences between DV518 and DV413 strains and between p518-L and p518-S strains. Residues are shown with H5 numbering system.

### The two purified DV518 (p518-L and p518-S) strains showed enhanced binding to MDCK cells

**Fig 5:**
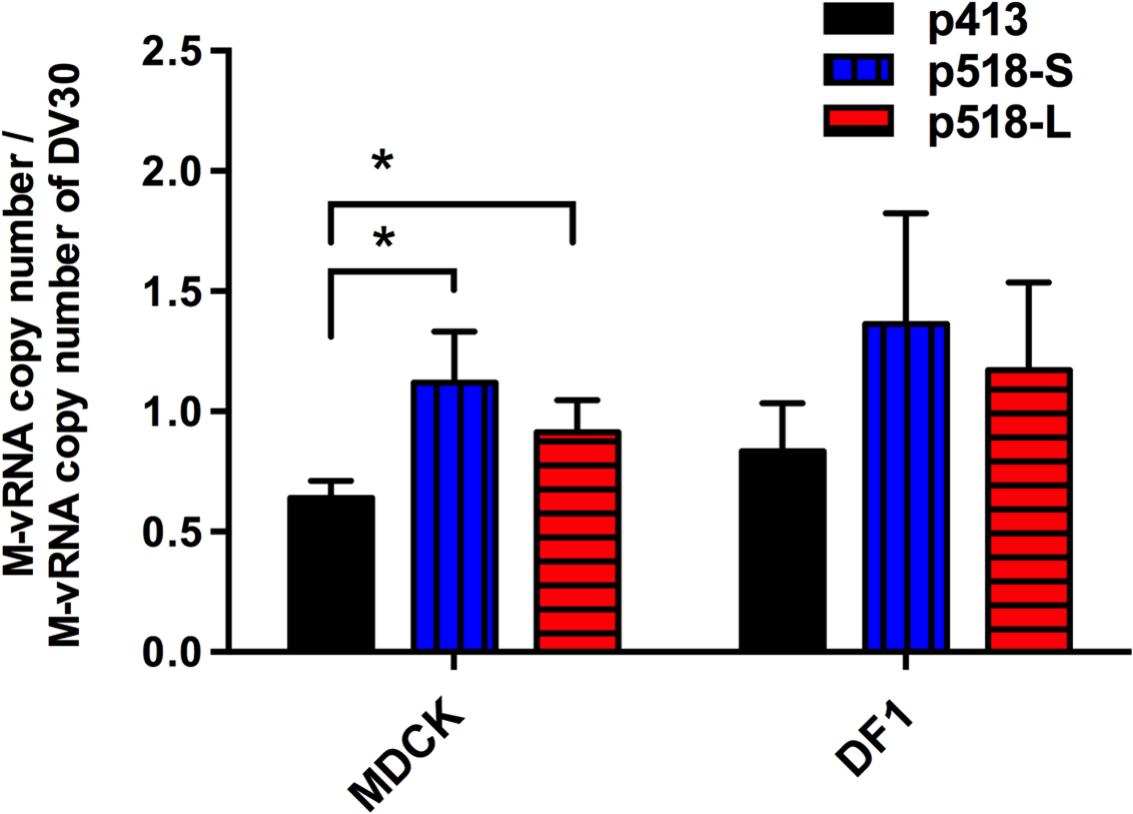
Cell binding ability of p518-L, p518-S and p413 virus strains to MDCK and DF1 cells. MDCK and DF1 cell monolayers were infected with p518-L, p518-S and p413 viruses at the same copy number and incubated at 4°C for 1 h. After extensive wash, viral RNAs were extracted from the infected cells and influenza viral M RNAs were quantified by quantitative RT-PCR. The copy numbers of M RNA attached to the cells were normalized to that of DV30 in each set of data. Results are shown as means with standard deviations in three experiments. *p<0.05. RT-PCR: Reverse transcription-polymerase chain reaction.

HA proteins, playing a predominant role in receptor binding, influence host range [13], virulence [29], and transmissibility [30, 31] of influenza viruses. Both p518-L and p518-S strains had Asp residue at position 170 (170D) in HA that is different from the 170Asn (170N) in p413. It is possible that the substitution of N170D in HA has the potential to modulate viral binding to the cells. We thus investigated the ability of these viruses to bind to the MDCK and DF1 cells. To perform virus binding assays, MDCK or DF1 cells representing different receptor conformations [32, 33] were incubated with the four duck H5N2 viruses [p518-L, p518-S, p413, or DV30-2 (our earliest duck H5N2 isolate with HA-170Asn)] for comparison. The DV30 virus with a receptor specificity previously characterized by glycoarray as α2,3 preference (Chung-Yi Wu and Chi-Huey Wong, personal communication), the typical avian-type specificity [34, 35], was used to normalize the copy numbers of vRNA binding to the cells. After one hour’s incubation at 4°C and extensive wash, the cells were collected for vRNA quantification by quantitative RT-PCR. As shown in Fig 5, both p518-L and p518-S strains had significantly higher capacity to bind to MDCK cells than did p413 virus (p<0.05). In addition, these two p518 strains also had higher capacity to bind to avian DF1 cells than did p413, though the difference among them was insignificant, indicating their common specificity to α2,3 receptors. All these results suggest that the substitution of N170D in HA may have the potential to modulate viral binding to MDCK cells, contributing to the better growth of DV518 and its derived two plaque-purified strains in mammalian cells.

### The p518-L virus strain showed higher polymerase activity in 293T cells

**Fig 6:**
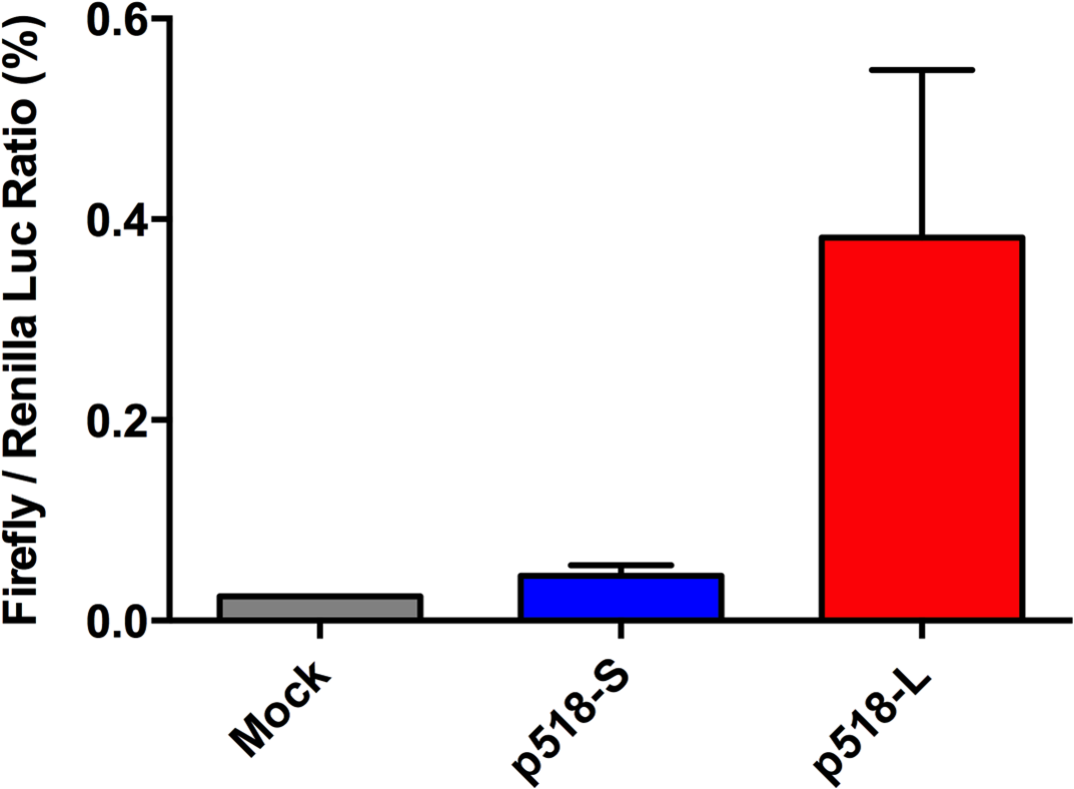
The viral polymerase activity of p518-S and p518-L strains in MDCK cells measured by minigenome assays. The viral polymerase activity of p518-S and p518-L strains was measured by minigenome assays as described in Materials and Methods. Data are presented as the ratios of firefly luciferase signal to Renilla luciferase signal, with means and standard deviations in triplicate experiments.

The p518-L and p518-S strains differed in three amino acids in three proteins of PB2, PA and M1. PB2 and PA are the components of viral polymerase that functions in viral transcription and replication [12]. We thus investigated whether p518-L and p518-S exhibited different polymerase activity in mammalian 293T cells. To assess possible differences in viral polymerase activity of these two p518 strains, a polymerase reporter plasmid and a Renilla-luciferase- expression plasmid (which serves as a transfection efficiency control) were co-transfected into 293T cells. These cells were then infected with either p518-L or p518-S virus, and the luciferase activity was evaluated at 9 h p.i. As shown in Fig 6, p518-L exhibited significantly higher luciferase activity than p518-S in 293T cells, suggesting that the former virus had higher polymerase activity than the latter virus.

**Fig 7:**
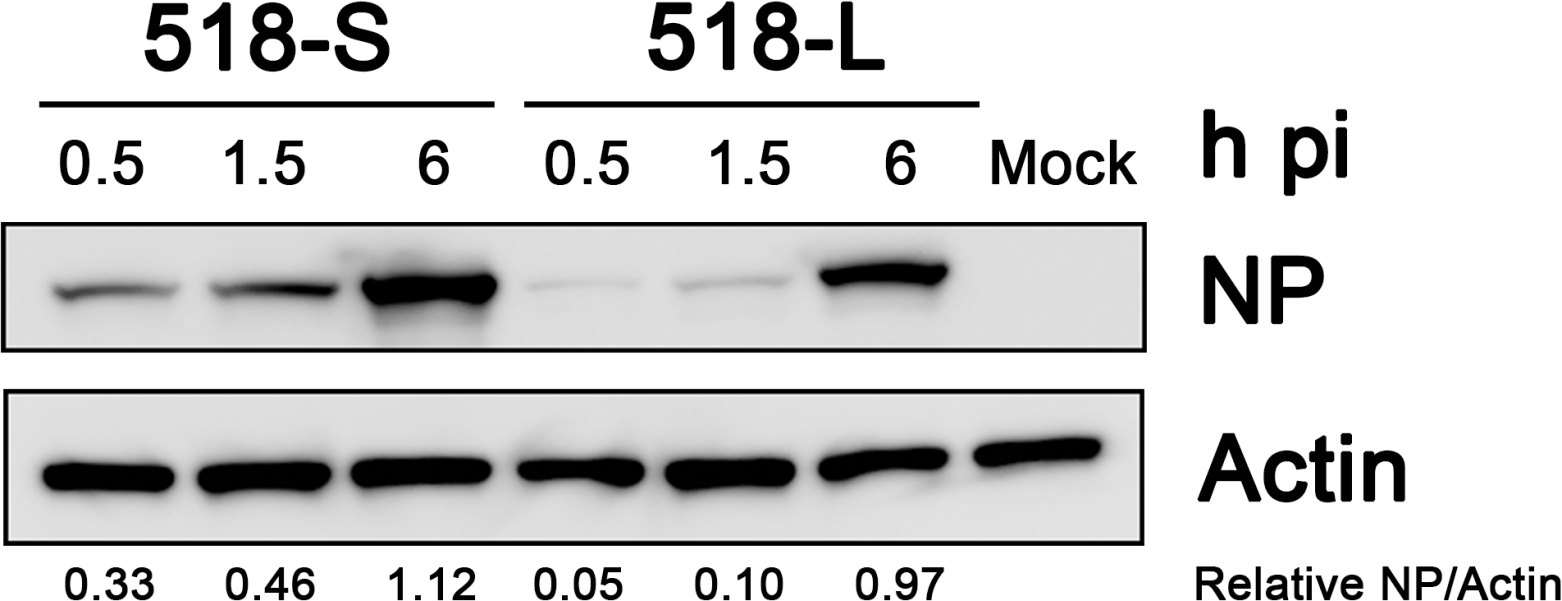
Evaluation of viral entry efficiency for p518-S and p518-L virus strains in 293T cells. The 293T cells were infected with p518-S and p518-L viruses at an MOI of 2. Cell lysates were collected at 0.5, 1.5 and 6 h p.i. and were examined by Western blot using anti-NP and anti-actin antibodies. Levels of protein and relative ratios of NP/Actin were analyzed by software Image J. **MOI**: multiplicity of infection

To determine whether the difference in luciferase activity observed in Fig 6 is due to the difference in viral entry efficiency, we assessed the viral entry of p518-L and p518-S by examining the abundance of NP protein at early time points after infection. As shown in Fig 7, the abundance of NP protein at early time points (0.5 and 1.5 h p.i.) was lower in p518-L infected 293T cells than in p518-S infected 293T cells; importantly, comparable NP protein levels were detected in cells infected by these two strains at 6 h p.i. Therefore, the higher luciferase activity observed in 518-L-infected 293T cells is indeed due to its greater polymerase activity but not due to its entry efficacy.

## Discussion

Poultry ducks carrying LPAI viruses have played critical roles in virus evolution [14, 15], including the novel H5N1 viruses found in Hong Kong that have impacted human health since 1997 [11]. However, few viral replication studies focus on duck LPAI H5N2 viruses. In this study, we characterized the phenotypic variations between the two genetically close duck LPAI H5N2 influenza viruses and revealed three major findings that helped us better understand the epidemiological aspects of influenza. First, variations of LPAI H5N2 viruses involved both intra-host and inter-host levels in ducks that may increase viral diversity, population dynamics, and continuous evolution. Second, intra-duck variations (shown in p518-L vs p518-S) included three residue substitutions in PA (P224S), PB2 (E72D), and M1 (A128T), whereas inter-duck variations (shown in DV518 vs DV413) contained the other three mutations in HA (N170D), NA (I56T), and NP (Y289H). These differences indicate that two levels of viral variations were involved in the mutations of different genes. Third, phenotypic variations of duck influenza p518-L strain with better replication in MDCK cells (compared to p518-S and p413) imply that duck-LPAI viruses may have a stronger potential to infect mammalian hosts under certain epidemiological settings [30, 36, 37]. Taken together, these findings provide new insight on the genotypic and phenotypic variations of LPAI H5N2 viruses from an individual duck to the entire duck population, prior to the process of expanding epizootic potential, and infecting other hosts.

Identifying the molecular determinants involved in genotypic and phenotypic variations of duck influenza viruses can help to understand mechanisms for viral persistence in nature, inter-species transmission, and elevated pathogenicity and virulence leading to severe epidemics and pandemics [13]. AIVs in waterfowl (predominantly wild ducks), which are important viral maintenance hosts, vary in infection susceptibility, exit routes, levels of viral excretion, and shedding patterns among different wild duck species [38]. Generally, these natural hosts infected with AIVs are asymptomatic or have mild infections. Several species of wild and domestic ducks are “silent spreaders” serving as the “Trojan horses” for influenza viruses [39]. Most importantly, certain AIV strains have the capability to cross host range barriers and infect other species, including humans. However, the selection mechanisms of LPAI virus among poultry ducks, which bridge natural maintenance hosts and other host species, are not well understood. Our data showed that the duck DV518 strain can grow to titers significantly higher than the duck DV413 strain in both mammalian MDCK and avian DF1 cell lines, thus implying the existence of factors that may contribute to viral replication. Moreover, the three plaque-purified strains, p518-L, p518-S, and p413, exhibited more complicated replication profiles: p518-L and p518-S replicated more efficiently than p413 in MDCK cells but showed comparable efficiency to that of p413 in DF1 cells. Evaluations of viral cell binding were in accordance with the growth advantages of both p518-L and p518-S strains in MDCK cells. Furthermore, varied viral polymerase activities between these two strains in 293T cells suggest that the difference in viral replication may be conferred by polymerase activities in host-specific cell lines. These findings not only support the importance of routine virological surveillance to understand and identify the phenotypic diversity of duck influenza viruses isolated from the same and different ducks, but also provide important information about virus evolution in both individual and population levels.

For intra-host variants, p518-L showed significantly higher polymerase activity in 293T cells when compared to p518-S. Detailed sequence analysis indicated that these two variants differed only at three positions (Table 1), and two of them are located in the components of the viral polymerase complex. These data indicate that the replication advantage of the AIV variants with minor genetic variation can be selected for within the same duck. How the amino acid variations in PB2, PA, and M1 contribute to the higher polymerase activity in p513-L is unclear. The minigenome assay was conducted at 9 h p.i. (a single replication cycle). This would exclude the possible involvement of mutation in M1, a protein known to play a role in vRNP export [40]. Position 72 in the PB2 is located in the PB2-N1 subdomain buttressing the PB1 thumb domain [41]. However, it is less likely that the mutation (from glutamate to aspartate without changing the charge) in p513-L would affect the function of the PB2 protein significantly. In PA, the mutation of S224P with other amino acid substitutions has been shown to increase the replication of duck H5N1 viruses in duck primary embryo fibroblast cells [42], and elevate viral virulence in a mouse model [42]. Whether the change at position 224 in PA (from proline to serine) may increase the polymerase activity of p513-L virus requires further investigation.

By contrast, inter-host variations of the two Taiwan-H5N2 DV518 and DV413 viruses were isolated from fecal droppings between poultry ducks (collected on July 28 and September 7, 2006 at a wholesale LPM (16) where ducks came from Yilan and Yunlin counties). These two viruses involved another three amino acid changes (N170D, I56T, and Y289H) in HA, NA, and NP, respectively [43–45]. DV518, p518-L and p518-S strains all have a 170D residue in HA in contrast to a 170N in DV413 and p413. Our data indicated that p518-L and p518-S viruses bind to MDCK cells, which possess both α2,6 and α2,3 receptors [33], more efficiently than the p413 virus (Fig 5). Furthermore, these three virus strains showed no significant difference in binding to A549 and DF1 cells (S5 Fig), which possess predominantly α2,6 and α2,3 receptors, respectively [32, 33], indicating that the receptor specificity of these viruses may not have been altered. Position 170 (or 158 in H3 structural numbering) is located at the tip of the HA (S6 Fig), near the receptor binding domain (RBD) in the globular head of HA1 [46]. This N170D (or N158D) mutation in HA has been correlated with better aerosol transmissibility of H5N1 viruses in ferrets [31] and guinea pigs [47] as well as human H5N1 cases in Vietnam and Egypt [48, 49]. Moreover, a quail-adapted duck H3N2 virus (i.e. virus adaption in **terrestrial birds** from aquatic birds) possessing HA-170D mutation replicated more efficiently in MDCK cells, even though these viruses had receptor specificities similar to those of the parental duck virus, and exhibited the potential to infect human cells *in vitro* and *ex vivo* [36]. The possible mechanism of this selection advantage might be attributable to the role of aspartate in increasing the negative charge on the surface of HA (a marker for adaptation to cultured mammalian cells) [50] (S6A and S6B Fig illustrate the polarity change of HA). Taken together, these results and findings by others suggest that N170D of HA does not alter the receptor specificity but may enhance its receptor-binding affinity by forming a more stable structure [31, 50, 51]. In other words, both p513-L and p513-S show enhanced binding to MDCK cells and the substitution of N170D in HA has the potential to modulate viral binding to MDCK cells. Whether the N170D mutation does contribute to the better binding of p518-L and p518-S to MDCK cells requires further investigation by using reverse genetics methods.

NA is the sialidase on a viral membrane [52], known to be associated with virion release and dissemination [53, 54]. It has been shown recently that NA can modulate receptor binding [55, 56]. However, position 56 in NA is located in the stalk region of the glycoprotein embedded in the membrane, not in the enzymatically active region interacting with sialyglycans [52, 57]. It is less likely that I56T mutation in NA would influence the binding of p513-L and p513-S to MDCK cells. NP protein is involved in resistance to the interferon-induced antiviral factor, Mx [58, 59] and cellular immunity [60, 61]. Several mutations in NP, including Y289H (located in the region predicted to interact with PB2 at the NP-NP interaction site) [62, 63], were recently identified in adaptation of the novel 2009 H1N1 virus to humans [64, 65]. These NP mutations also allow the H5N1 virus to escape from human MxA by inhibiting the host innate immune response to Mx1 [65]. DV518, p518-L and p518-S possess the 289H in NP. This may make them become more resistant to the host’s antiviral strategies. Whether HA (N170D), NA (I56T), and NP (Y289H) mutations alone or in combination contribute to the growth advantage of DV518, p518-L, and p513-S in MDCK cells requires further investigation by using reverse genetics method.

The evolution of influenza viruses is a continuous process of interactions between viruses and hosts. Due to the infidelity of viral RNA-dependent RNA polymerase and large population sizes, many different genetic alterations may occur during RNA genome replication. Among these heterogeneous viral populations, minor viral variants may have significant implications for quasispecies evolution [66]. Our duck LPAI H5N2 viruses had slow evolution and stable viral sequences, because the viral sequences remained the same after one passage in chicken eggs and through several steps of plaque-purification (data not shown). However, the selection of AIVs does occur in poultry ducks, because neither p518-L nor p518-S is identical to the original DV518 isolate in full-genome sequences. This result indicates that the overall virus population derived from the field is a mixture of heterogeneous sub-populations of viral variants. Our finding on intra- and inter-duck phenotypic variations of the viruses which occurred at a cellular level is consistent with previous studies from duck samples demonstrating varied biological characteristics [14] and LPAI H1N9 and H6N1 isolates from wild birds replicates in ferrets [67]. For inter-duck variants, DV518 had growth advantage over DV413. For intra-duck variants, a minor sub-population of variants such as p518-L may have growth advantage in mammalian cells, causing health problem in humans after further evolution.

There are four major limitations to this study. First, the viral mutations identified from this study need further investigation, using reverse genetic approaches to determine whether a single mutation or multiple co-mutations were required to enhance viral replication in mammalian cells. Moreover, whether DV518 and its derivative viruses could multiply more efficiently than DV413 and its derivative virus in mice or other primates needs to be investigated. Second, the original samples amplified in the SPF eggs may not represent the viral population in the host [17]. Third, the two genetically close duck H5N2 viruses we studied cannot reflect the whole of viral population dynamics and selection in ducks, nor the previously reported H5N2 viruses in chickens in Taiwan [13]. In reality, whether the studied duck viruses have other additional unidentified subpopulations remains unclear. Fourth, the roles of host or environmental factors or concurrent infection of multiple subtypes of AIVs in intra- or inter-host evolution of duck H5N2 viruses deserve further investigation. Fully understanding the spectrum of viral variants (various percentages of the subvariants) and their roles in viral phenotypic variations through selection and fitness with trends in becoming dominant subvariants requires more modernized laboratory methods (such as reverse genetics, next generation sequencing) to provide stronger conclusion.

In summary, this study reveals several molecular determinants that may enhance cell binding and replication of duck LPAI H5N2 virus in MDCK cells. As anti-H5N2 seropositive poultry workers were detected [68], molecular markers for increasing viral replication as well as higher ability to inhibit antiviral immunity will be helpful in selecting the best strain for a future cell-based human H5N2 vaccine. The higher binding to and the greater replication capability of DV-518 (particularly DV-518-L) in MDCK cells implies that duck influenza virus sub-variants may possess potential for inter-species transmission to mammalian hosts. Such mutation events involve a series of host adaptations and selection, which may result in a novel virus with increasing replication efficiency, pathogenicity, and pandemic potential, as recently observed with novel H5N8 AIVs causing poultry outbreaks worldwide [69, 70]. Taken together, the integrated information from the micro-evolution of the virus to population dynamics can provide us with better clues about various selection mechanisms involving route of infection, receptor binding, replication efficiency, virion packaging, herd immunity, and efficient transmissibility [71]. In view of the most recent severe and fatal human cases caused by novel LPAI H7N9, H6N1 and H10N8 viruses [6, 7, 72], there is an increasing need to gain more knowledge about molecular determinants associated with host range and inter-species transmission of LPAI viruses. Therefore, efforts to establish integrated surveillance in areas involving wild birds, poultry ducks, chickens and mammalian hosts are urgently needed. By using whole-genome, deep pyrosequencing analysis and phenotypic characterizations, the evolutionary features of AIVs at a sub-population level after passaging through either avian or mammalian cells can be used to identify host-specific molecular markers associated with alterations in viral replication [61, 73]. A series of studies focused on an H7N9 drug-resistant sub-population and their fitness *in vivo* can serve as a good example [74, 75]. This effort will help us fully understand the selection mechanisms and dynamic changes of molecular determinants in different host species at both micro- and macro-levels, leading to improved risk assessment, vaccine development and pandemic preparedness.

## Supporting Information

S1 Fig Plaque morphologies of DV518, DV413 and their plaque-purified strains (p518-S, p518-L, p413) on MDCK cells. MDCK cells were infected with DV413, DV518, p413, p518-S and p518-L virus strains. After incubation at 37°C for 72 h, cells were fixed by paraformaldehyde and stained with crystal violet. Mean plaque area (mm^2^) of plaque in a representative well for each strain was analyzed by software Image J, and was shown as mean ± standard deviations in the parentheses.
(PDF)

S2 Fig Plaque morphologies of p518-L and p518-S viruses shown by focus- forming assay on MDCK cells. MDCK cells were infected with p518-L or p518-S viruses. After incubation at 37°C for 48 h, infected cells were treated with a mixture of mouse anti-influenza M monoclonal antibody (Millipore) and mouse anti-influenza NS1 monoclonal antibody (Santa Cruz Biotechnology) after fixation. The HRP-conjugated anti-mouse secondary antibody (Jackson) was then added and the foci were subsequently developed by adding peroxidase substrate using a commercial kit (Vector Laboratories).
(PDF)

S3 Fig The growth kinetics of the three plaque-purified virus strains (p518-L, p518-S and p413) in MDCK cells as determined by TCID_50_ method. MDCK cells were infected with p518-L, p518-S, and p413 virus strains at an MOI of 0.01. The supernatants were harvested at the indicated time points. The TCID_50_ method was used to examine the viral growths. The results are shown as means ± standard deviations of triplicate samples.
(PDF)

S4 Fig Chromatogram comparing nucleotide sequences of HA, PA and NP in parental viruses (DV518 and DV413) and a series of plaque-purified viruses. Abbreviations: 518, DV518 virus; 413, DV413 virus; S-P1, S-P2, and S-P3, small-plaque virus obtained by the first, second, and third round of plaque purification of DV518, respectively; L-P1, L-P2, and L-P3, large-plaque virus obtained by the first, second, and third round of plaque purification of DV518, respectively; 413-P1, virus obtained by first-round plaque purification of DV413.
(PDF)

S5 Fig p518-L, p518-S and p413 virus strains showed no difference in binding to A549 and DF1 cells. A549 and DF1 cell monolayers were infected with p518-L, p518-S and p413 viruses at the same copy number and then incubated at 4°C for 1 h. After extensive wash, viral RNAs were extracted from the infected cells and influenza viral M RNAs were quantified by quantitative RT-PCR. The copy numbers of M RNA attached to the cells were normalized to that of DV30 in each set of data. Results are shown as means with standard deviations in three experiments.
(PDF)

S6 Fig Modeled surface polarities and position 170 in HA protein. Surface polarities of HA with positions of 170D (a) and 170N (b) are shown. Position 170 (or 158 in H3 numbering) of HA is at the top of the HA molecule (c). The orange region, HA 161–175, represents the residues changed to the amino acids possessed by DV518 or DV413 (d). All structures shown are based on the backbone of A/Viet Nam/1203 (pdb: 2FK0).
(PDF)
