# Supplementary figures and images for: Phenotypic and Genetic Characterization of Avian Influenza H5N2 Viruses with Intra- and Inter-Duck Variations in Taiwan
Source: PLoS One. 2015 Aug 11;10(8):e0133910. doi: 10.1371/journal.pone.0133910 (PMC4532476; doi:10.1371/journal.pone.0133910)

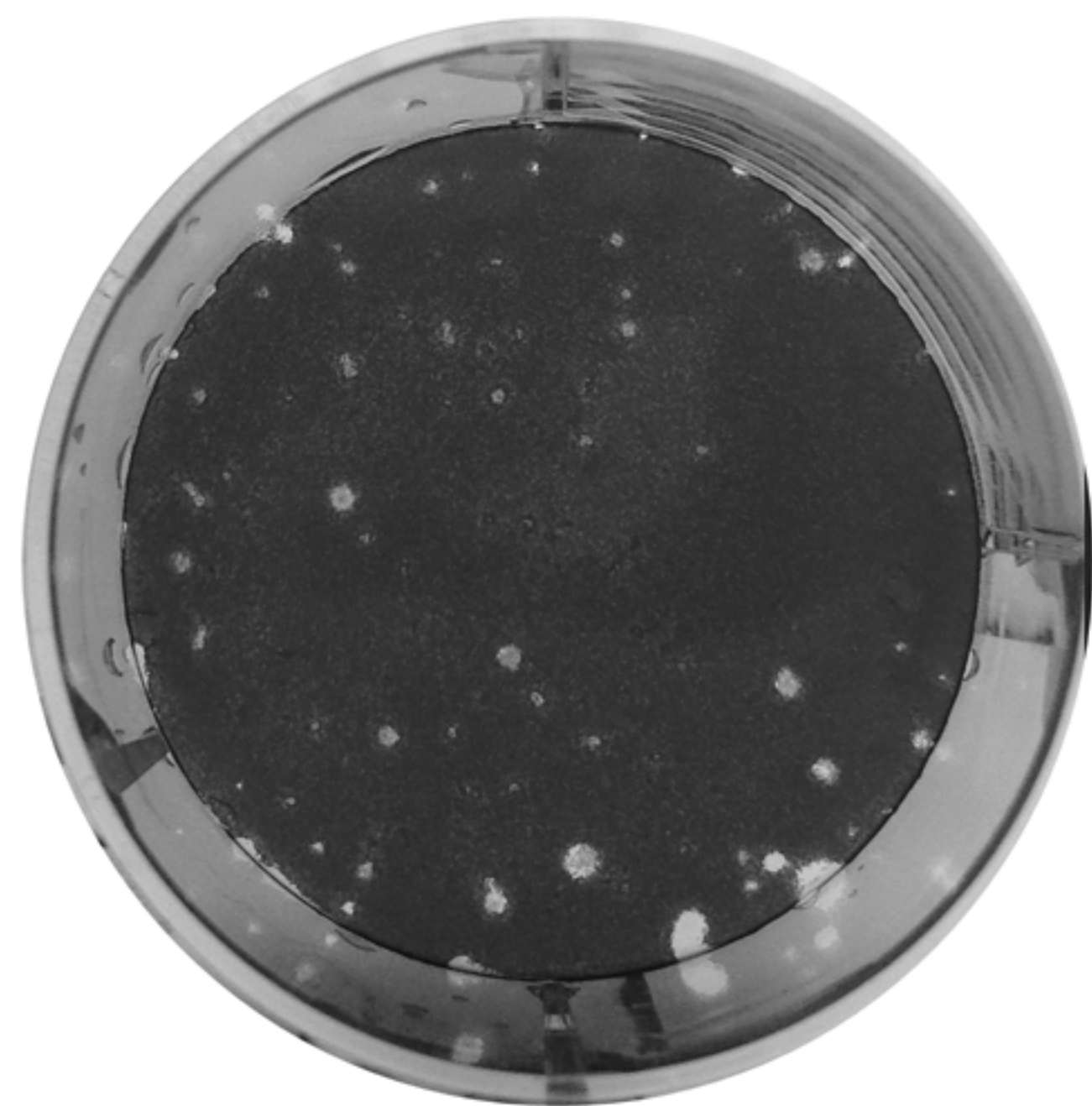

**DV413**  
**( $0.22 \pm 0.38$ )**

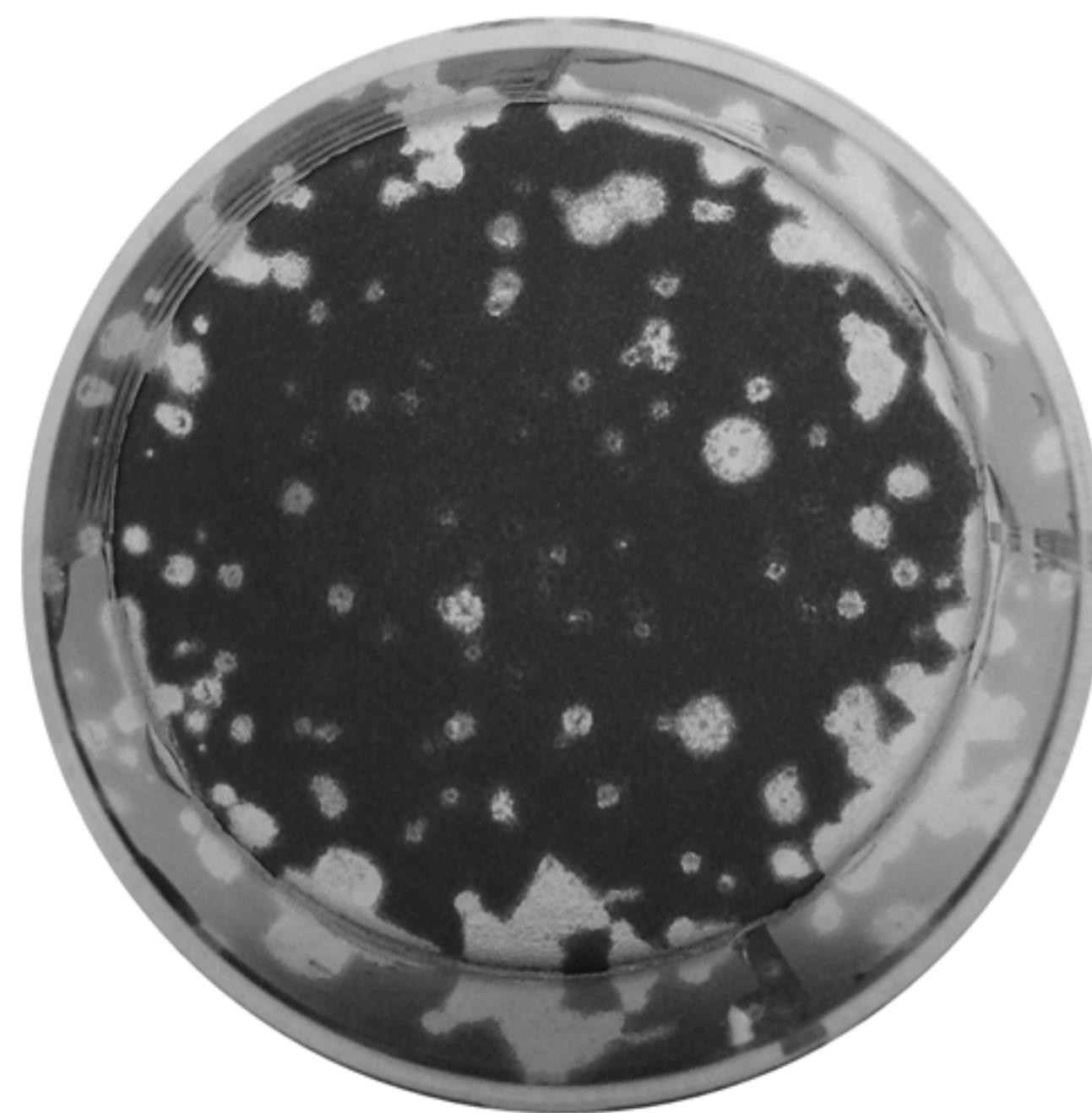

**DV518**  
**( $0.77 \pm 1.49$ )**

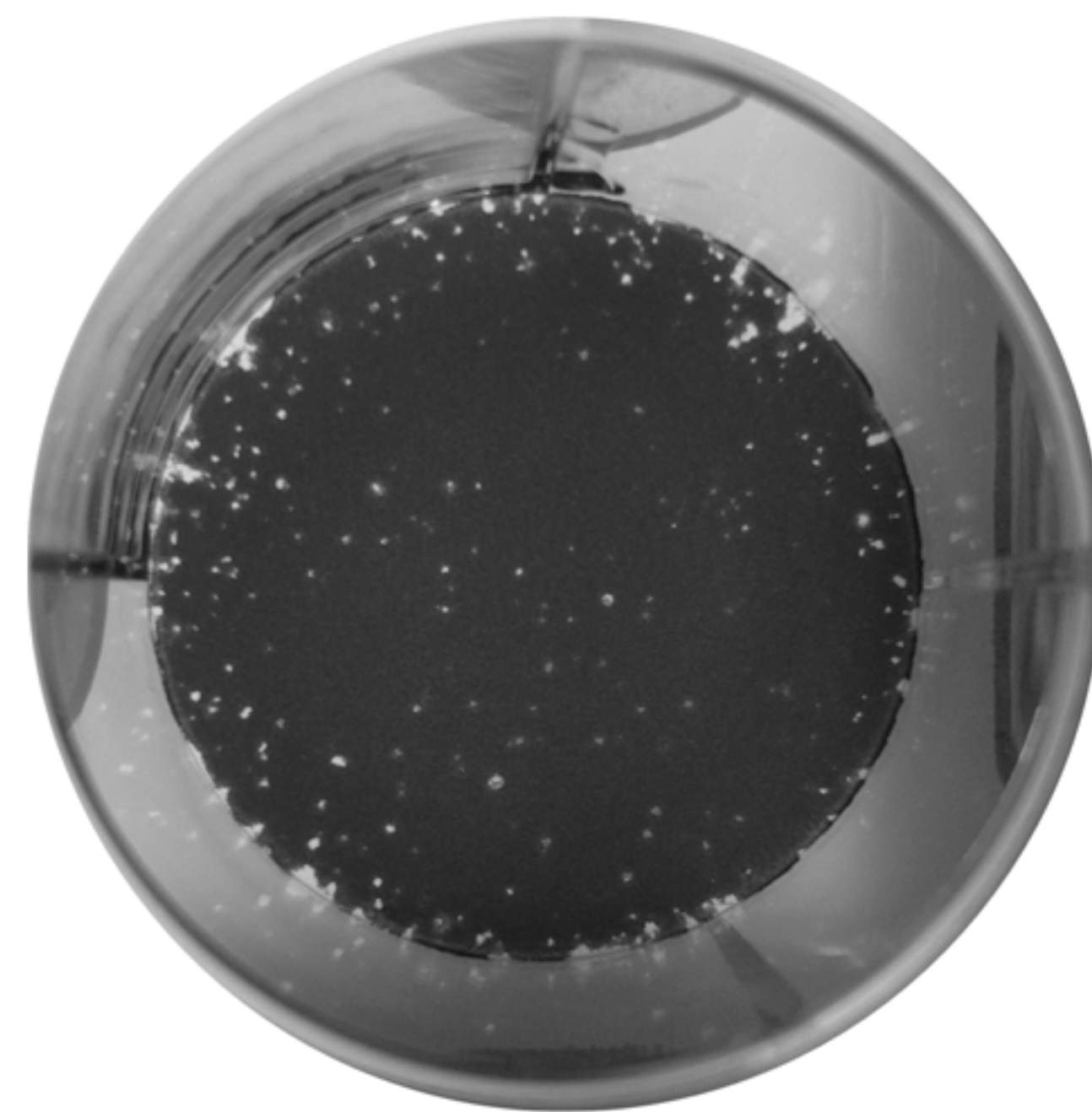

**p413**  
**( $0.10 \pm 0.10$ )**

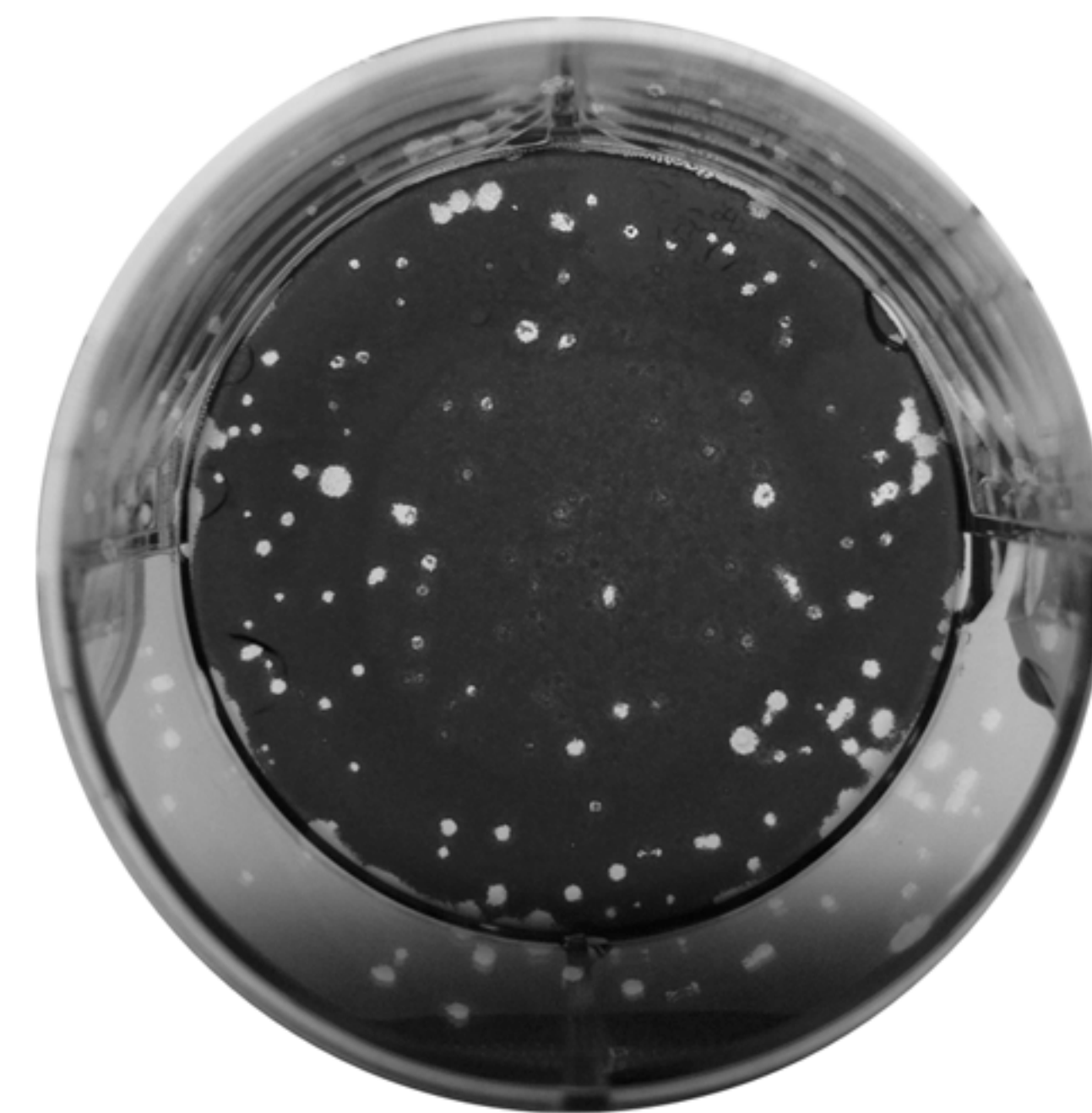

**p518-S**  
**( $0.29 \pm 0.30$ )**

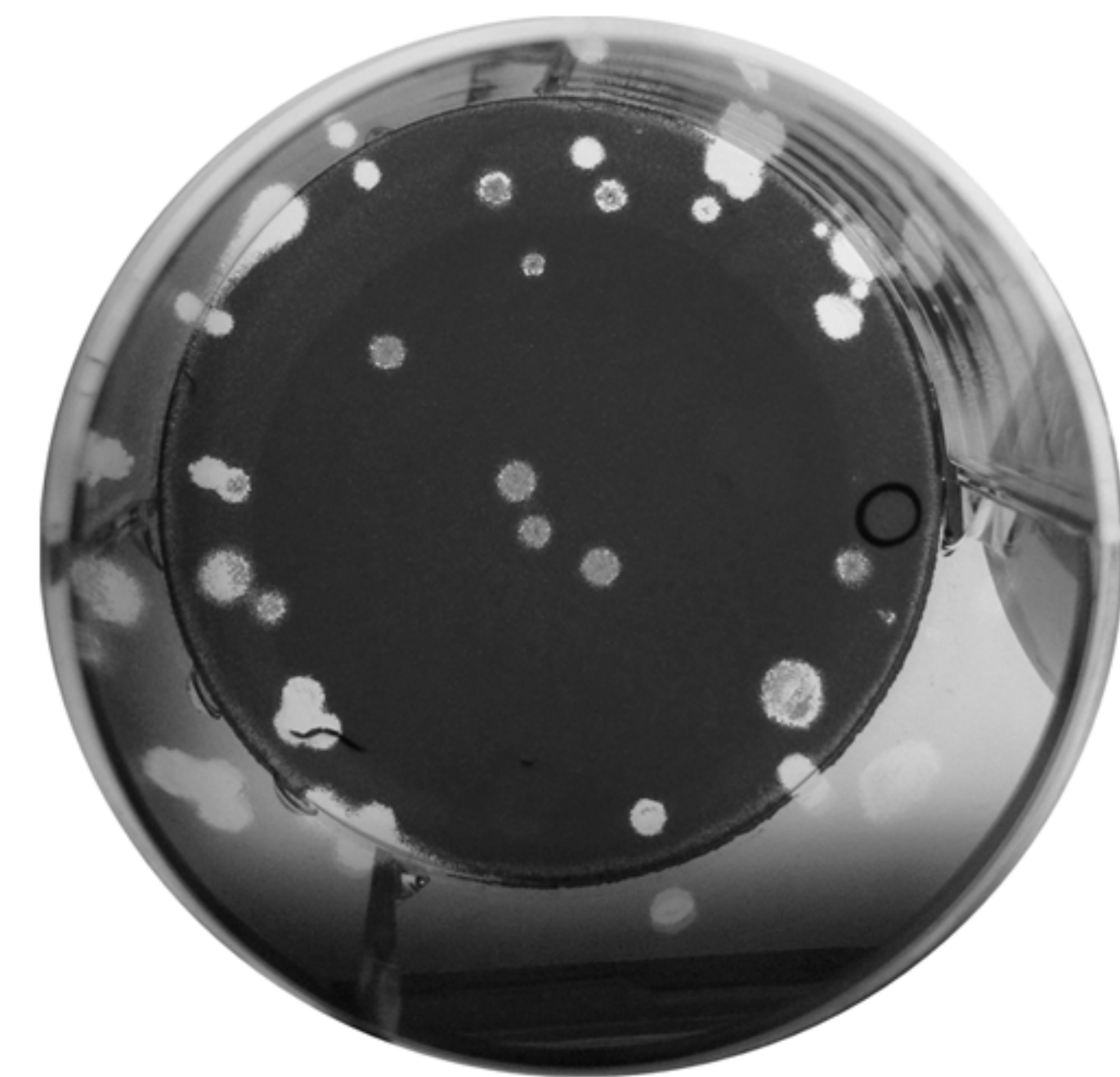

**p518-L**  
**( $1.66 \pm 1.55$ )      ( $\text{mm}^2$ )**

Supplement: S1 Fig — MDCK cells were infected with DV413, DV518, p413, p518-S and p518-L virus strains. After incubation at 37°C for 72 h, cells were fixed by paraformaldehyde and stained with crystal violet. Mean plaque area (mm2) of plaque in a representative well for each strain was analyzed by software Image J, and was shown as mean ± standard deviations in the parentheses. (PDF) [file pone.0133910.s001.pdf]

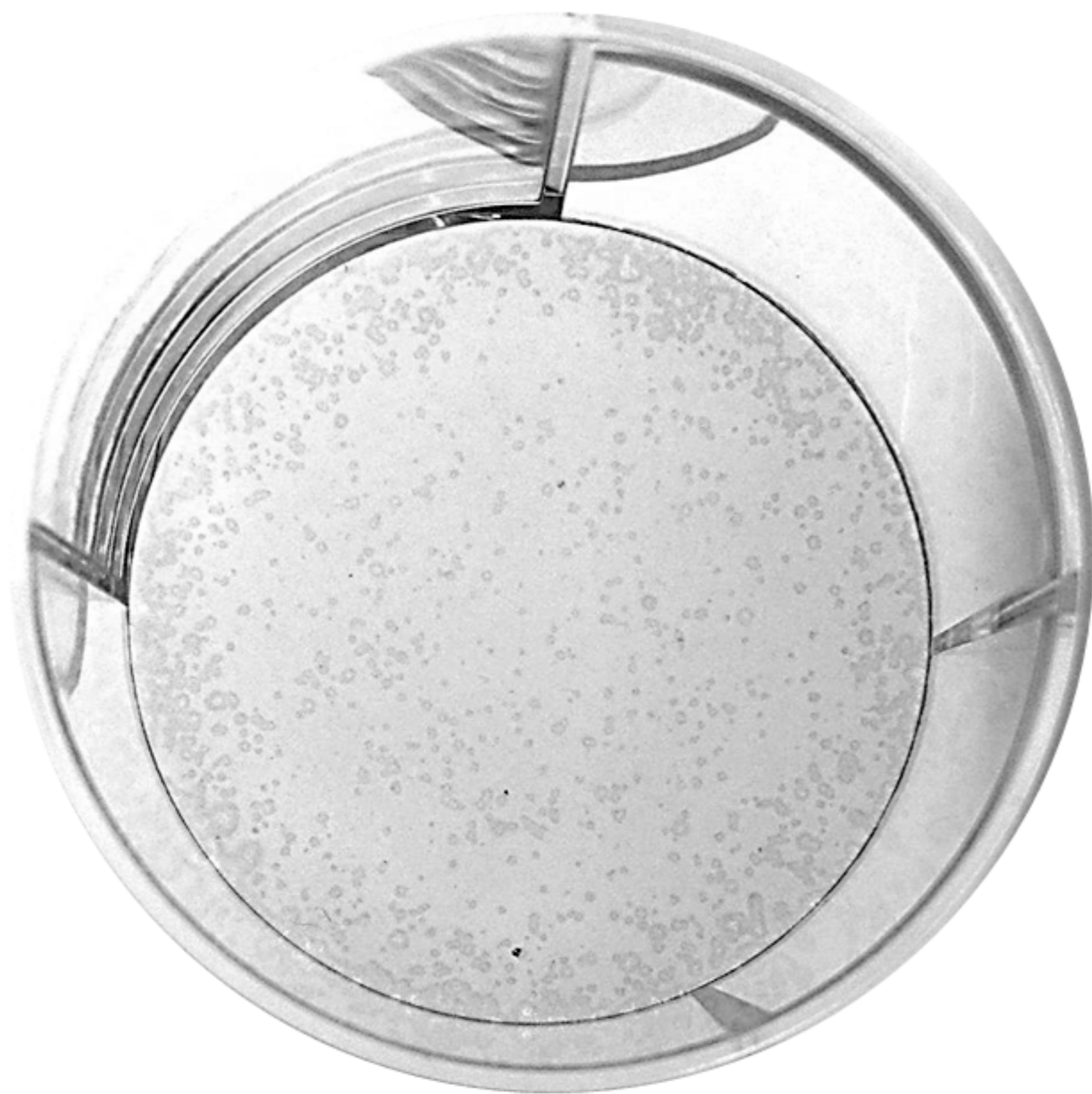

**p518-S**

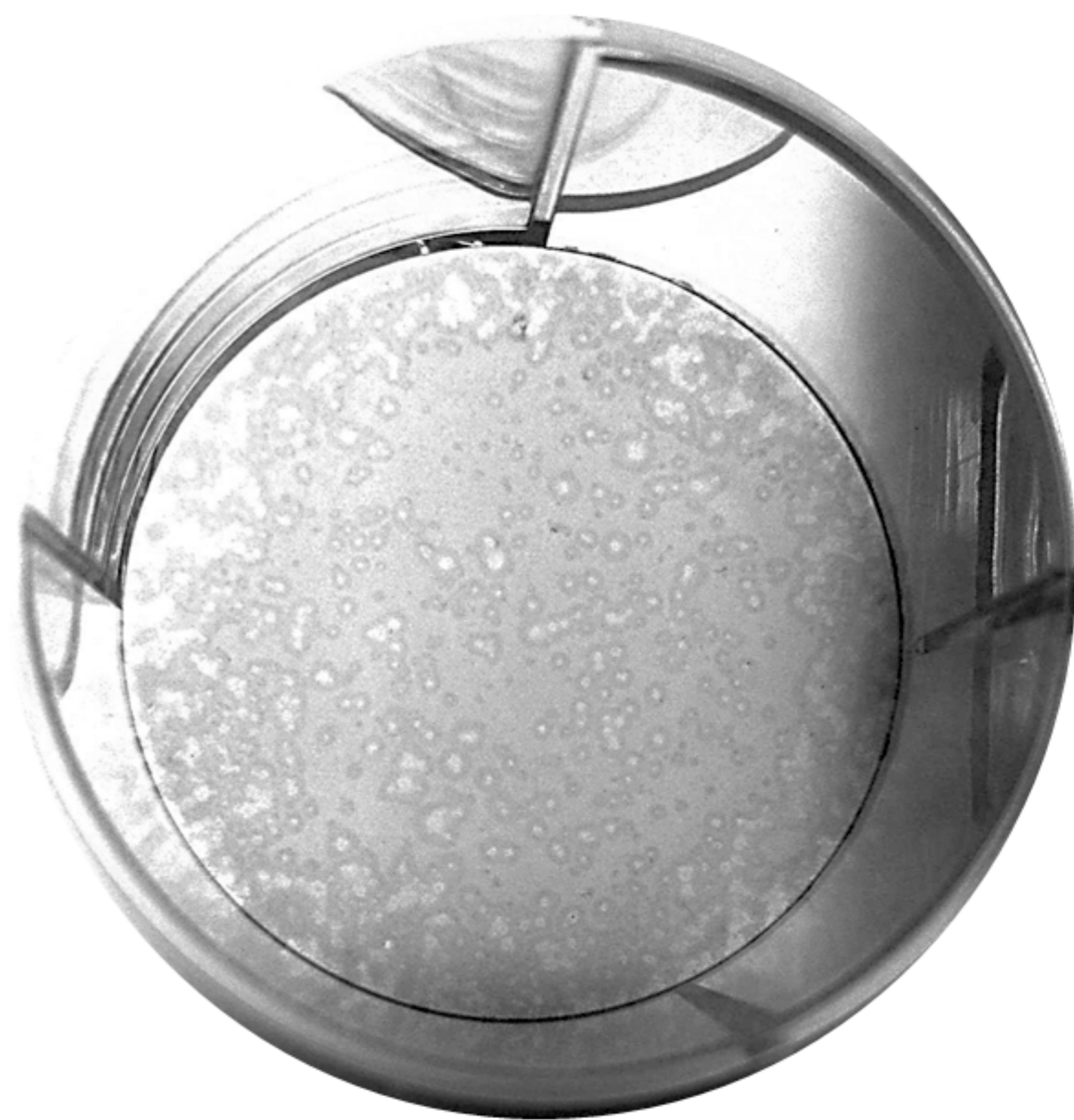

**p518-L**

Supplement: S2 Fig — MDCK cells were infected with p518-L or p518-S viruses. After incubation at 37°C for 48 h, infected cells were treated with a mixture of mouse anti-influenza M monoclonal antibody (Millipore) and mouse anti-influenza NS1 monoclonal antibody (Santa Cruz Biotechnology) after fixation. The HRP-conjugated anti-mouse secondary antibody (Jackson) was then added and the foci were subsequently developed by adding peroxidase substrate using a commercial kit (Vector Laboratories). (PDF) [file pone.0133910.s002.pdf]

**MDCK**  
**MOI=0.01**

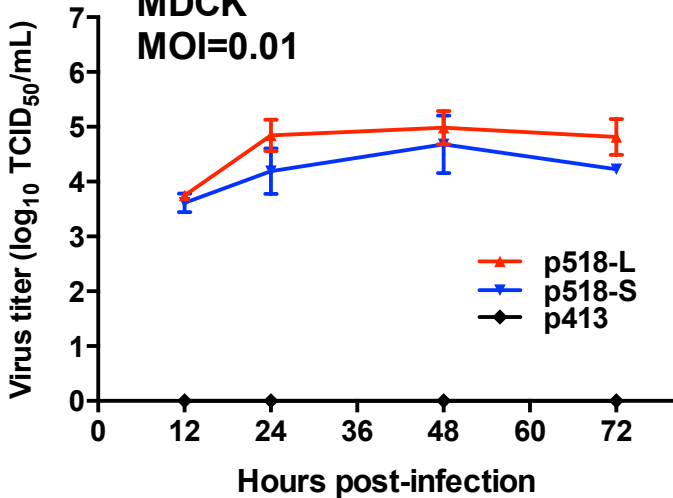

Supplement: S3 Fig — MDCK cells were infected with p518-L, p518-S, and p413 virus strains at an MOI of 0.01. The supernatants were harvested at the indicated time points. The TCID50 method was used to examine the viral growths. The results are shown as means ± standard deviations of triplicate samples. (PDF) [file pone.0133910.s003.pdf]

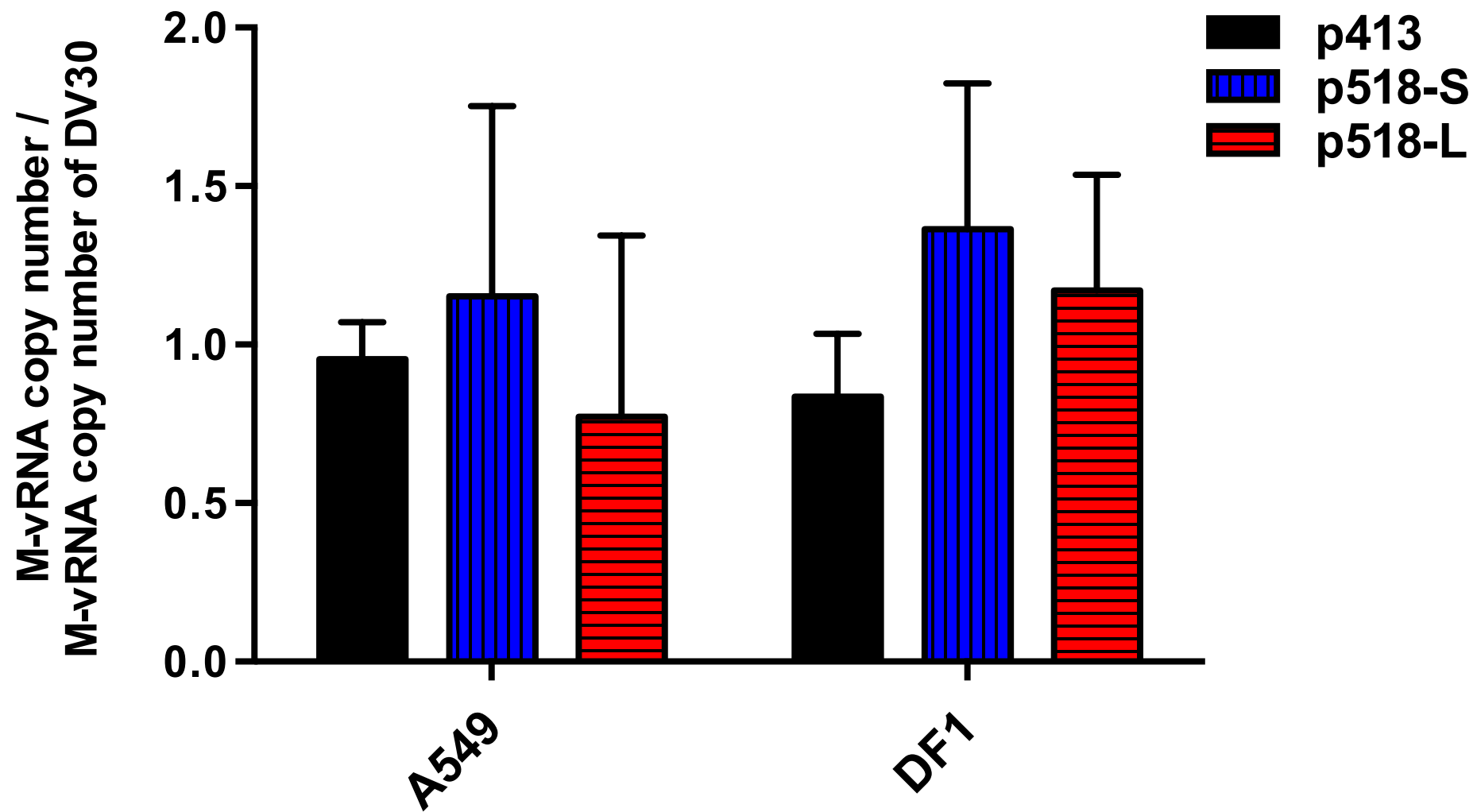

Supplement: S5 Fig — A549 and DF1 cell monolayers were infected with p518-L, p518-S and p413 viruses at the same copy number and then incubated at 4°C for 1 h. After extensive wash, viral RNAs were extracted from the infected cells and influenza viral M RNAs were quantified by quantitative RT-PCR. The copy numbers of M RNA attached to the cells were normalized to that of DV30 in each set of data. Results are shown as means with standard deviations in three experiments. (PDF) [file pone.0133910.s005.pdf]

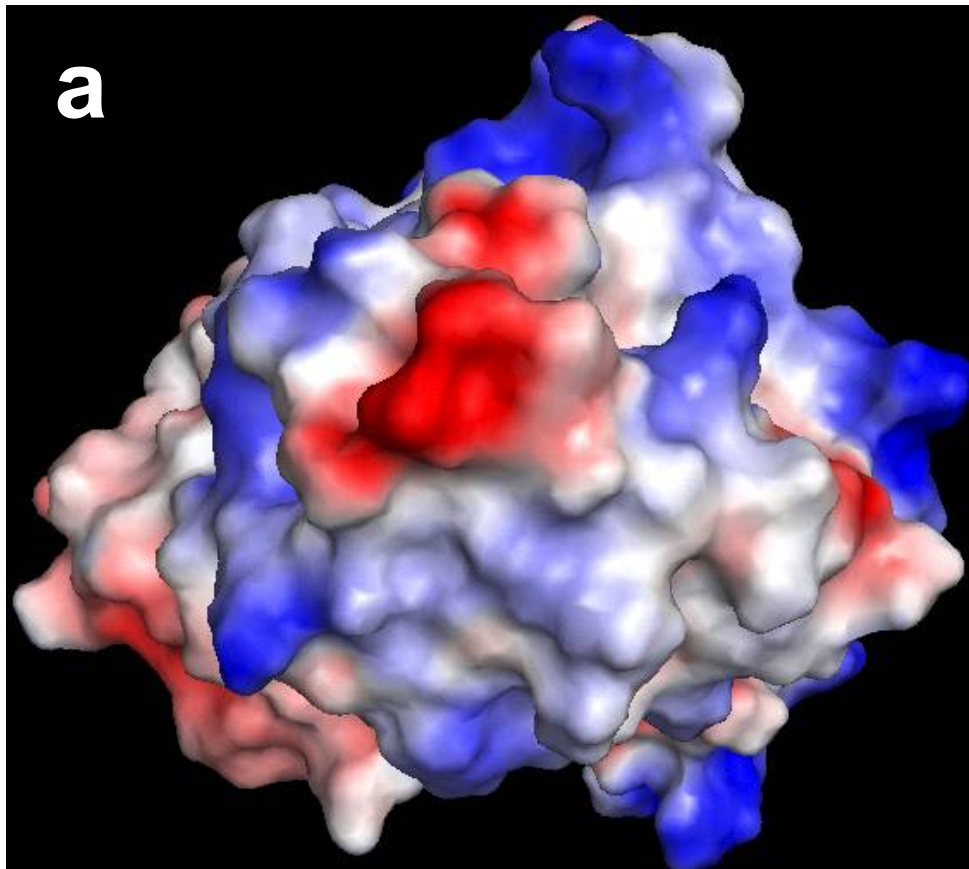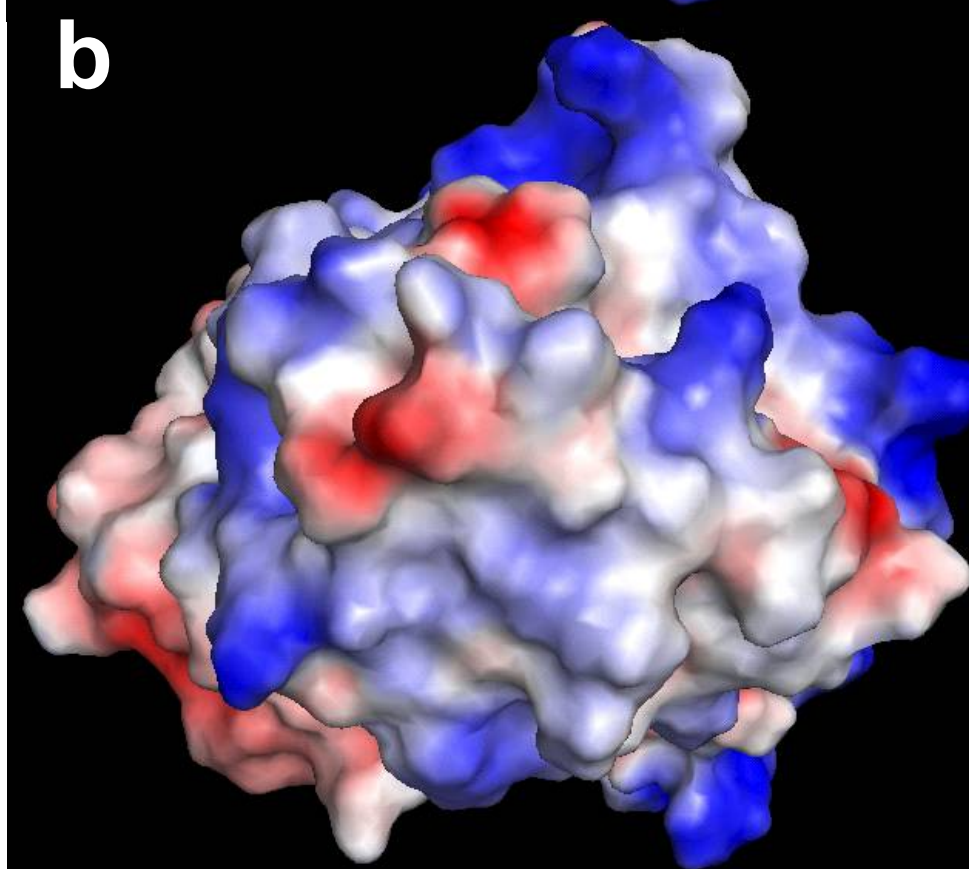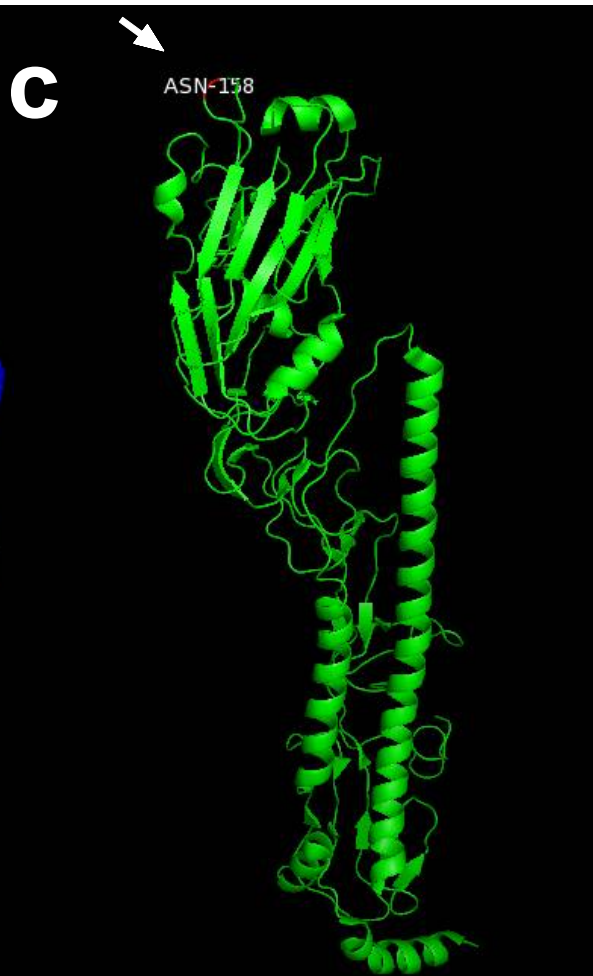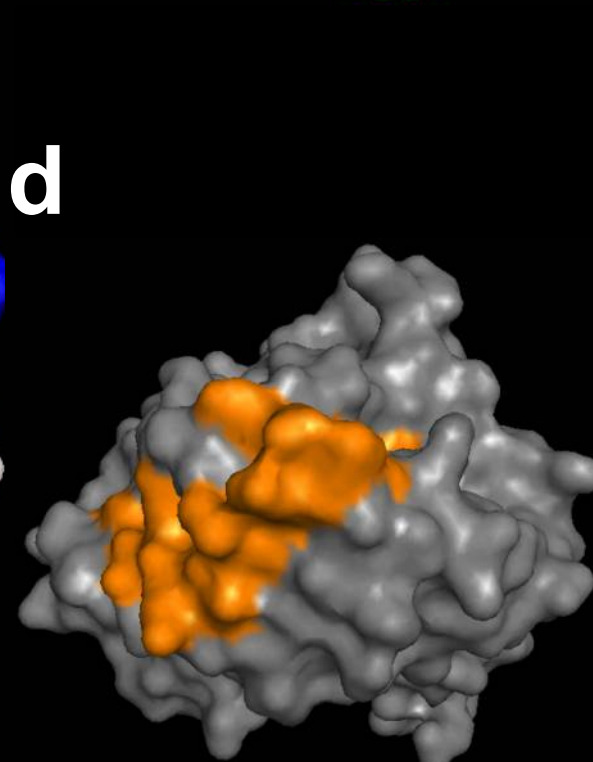

Supplement: S6 Fig — Surface polarities of HA with positions of 170D (a) and 170N (b) are shown. Position 170 (or 158 in H3 numbering) of HA is at the top of the HA molecule (c). The orange region, HA 161–175, represents the residues changed to the amino acids possessed by DV518 or DV413 (d). All structures shown are based on the backbone of A/Viet Nam/1203 (pdb: 2FK0). (PDF) [file pone.0133910.s006.pdf]
