# Supplementary material for: Phenotypic and Genetic Characterization of Avian Influenza H5N2 Viruses with Intra- and Inter-Duck Variations in Taiwan
Source: PLoS One. 2015 Aug 11;10(8):e0133910. doi: 10.1371/journal.pone.0133910 (PMC4532476; doi:10.1371/journal.pone.0133910)

HA 170: nucleotied substitution at position 508 (170D/170N)

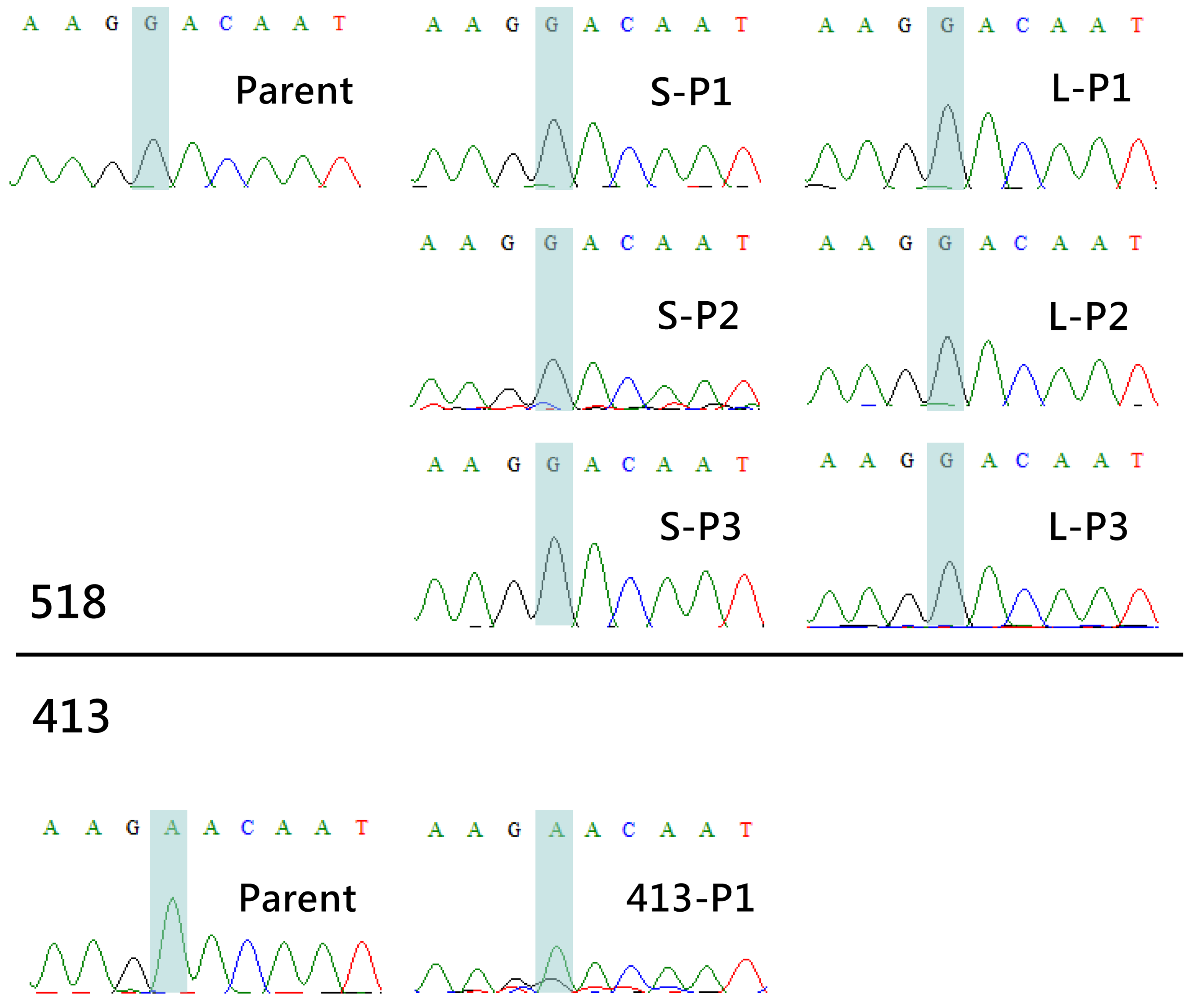

PA 224: nucleotied substitution at position 670 (224P/224S)

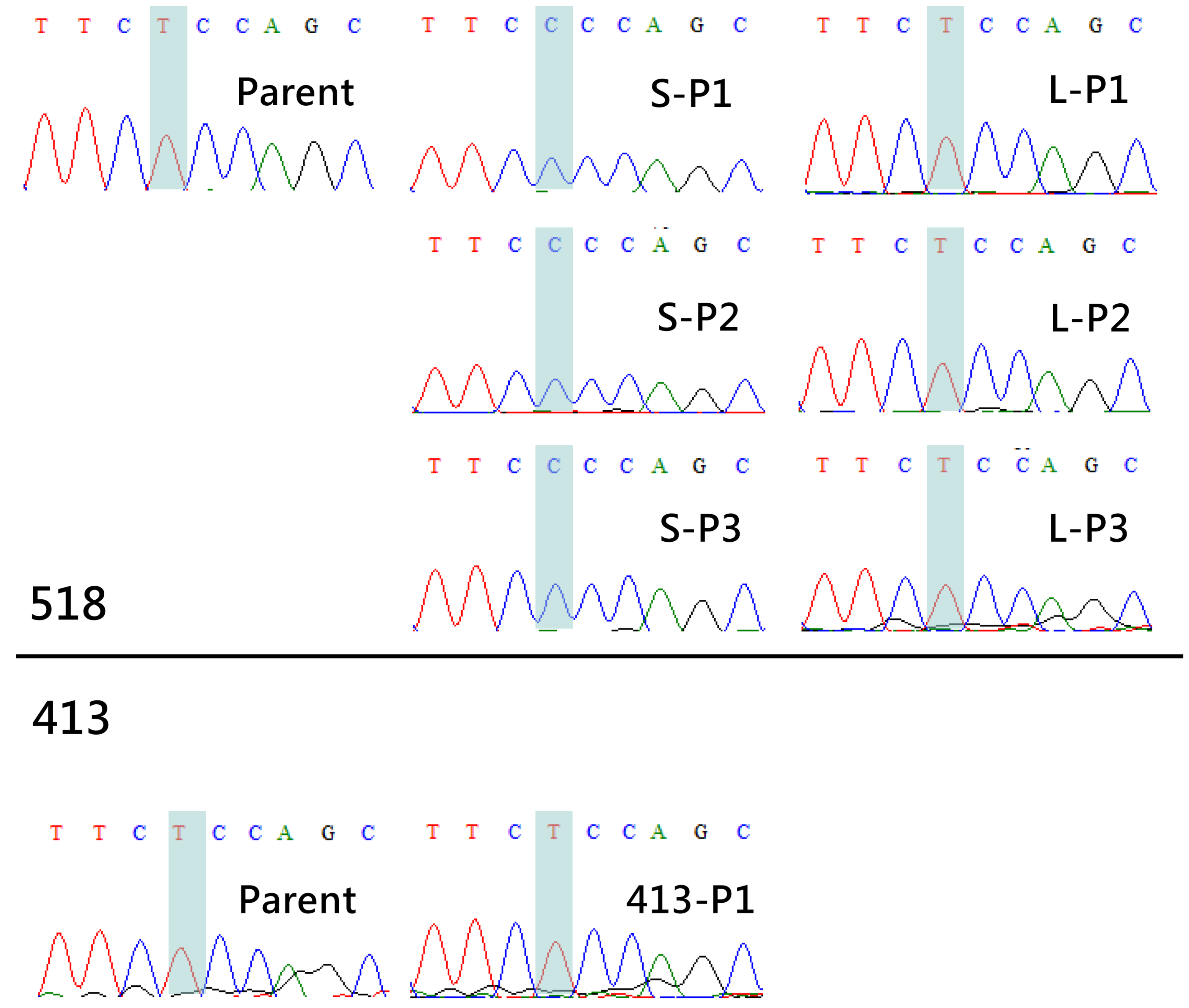

NP 289: nucleotied substitution at position 865 (289H/289Y)

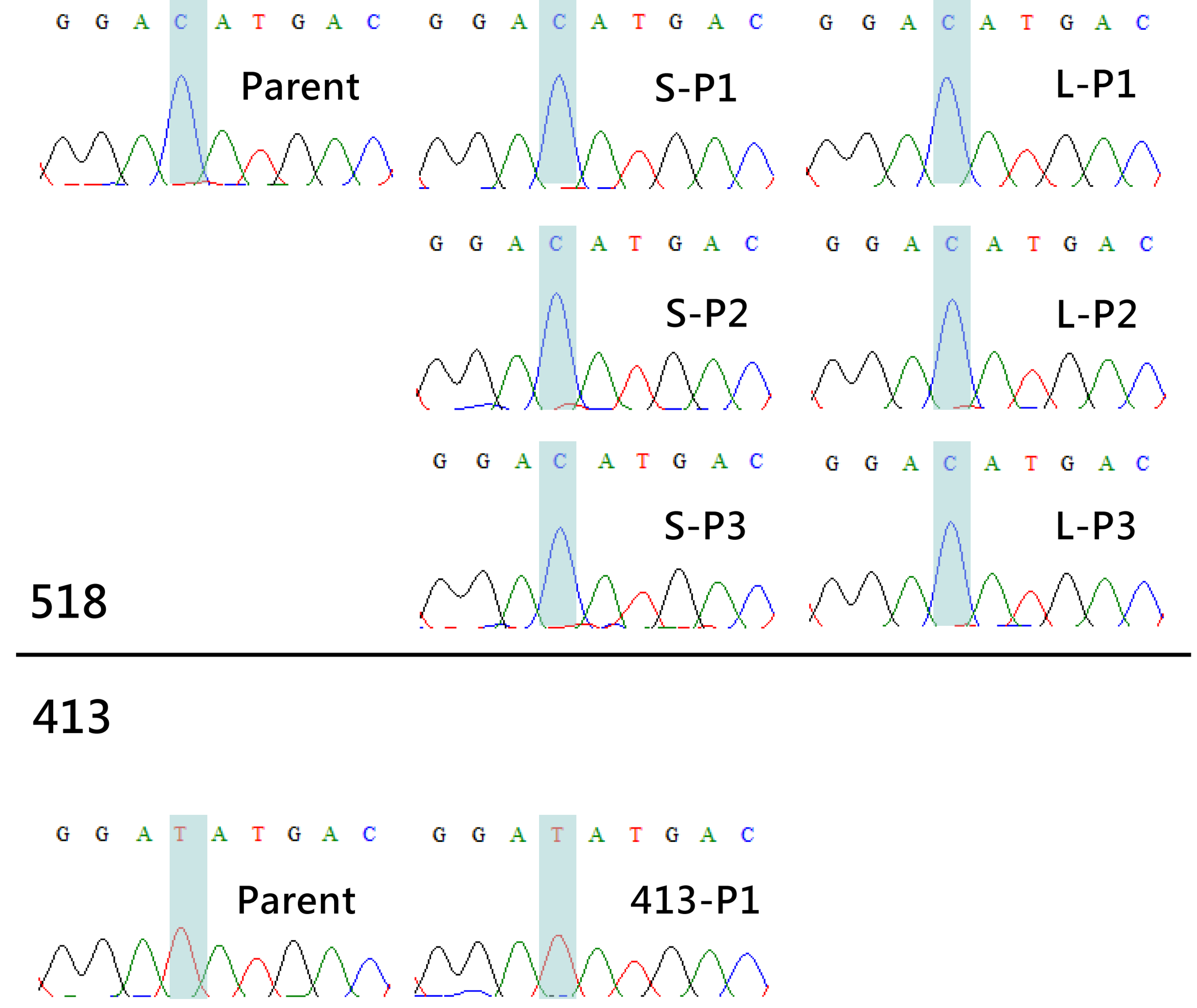

Supplement: S4 Fig — Abbreviations: 518, DV518 virus; 413, DV413 virus; S-P1, S-P2, and S-P3, small-plaque virus obtained by the first, second, and third round of plaque purification of DV518, respectively; L-P1, L-P2, and L-P3, large-plaque virus obtained by the first, second, and third round of plaque purification of DV518, respectively; 413-P1, virus obtained by first-round plaque purification of DV413. (PDF) [file pone.0133910.s004.pdf]
